# Evaluation of *Passiflora edulis* Seed Extract and Piceatannol in Attenuating Hyperglycemia and Oxidative Stress Using Human and Nonhuman Antidiabetic Enzymes, Human Neutrophil Oxidative Burst, and Embryonic Zebrafish Hyperglycemia Model

**DOI:** 10.1021/acsomega.6c03845

**Published:** 2026-09-14

**Authors:** Elaine L. S. S. Mendonça, Ana Ramalho, Boniek G. Vaz, Alane C. M. Oliveira, Jadriane A. Xavier, Marisa Freitas, Eduarda Fernandes, Marília O. F. Goulart

**Affiliations:** † Institute of Chemistry and Biotechnology, 28112 Federal University of Alagoas, 57072-900 Maceió, Alagoas, Brazil,; ‡ LAQV/REQUIMTE, Laboratory of Applied Chemistry, Department of Chemical Sciences, Faculty of Pharmacy, University of Porto, 4050-313 Porto, Portugal; § Chromatography and Mass Spectrometry Laboratory,Federal University of Goiás, Goiânia 74690-900, GO, Brazil; ∥ College of Nutrition, 28112 Federal University of Alagoas, 57072-900 Maceió, Alagoas, Brazil; ⊥ National Institute of Science and Technology in Bioanalytics Lauro Kubota (INCTBio-LK), University of Campinas (UNICAMP), 13083-861 Campinas, São Paulo, Brazil

## Abstract

*Passiflora edulis* seeds are frequently discarded as agro-industrial residues despite being a source of bioactive compounds such as piceatannol (PIC). Their valorization through ethanolic extraction aligns with circular economy principles and enables the investigation of antioxidant, anti-inflammatory, and antidiabetic potential relevant to metabolic disorders associated with oxidative stress, inflammation, and impaired glucose homeostasis. Therefore, this study compared the biological activities and toxicological profiles of *P. edulis* seed ethanol extract (PESE) and PIC using in vitro and in vivo assays, including human and nonhuman models, and hyperglycemia induced during embryogenesis. The zebrafish procedures were approved by the Animal Use Ethics Committee (CEUA; protocol No. 13/2022). LC–MS identified PIC among the main constituents of PESE, which exhibited a high total phenolic content and relevant antioxidant capacity. PIC showed greater antioxidant potency than PESE against DPPH^•^, HOCl, and O_2_^•–^. It also inhibited α-amylase and α-glucosidase, with IC_50_ values of 11.8 and 3.1 μg/mL, respectively, outperforming acarbose against α-glucosidase, and activated glucokinase. In contrast, PESE inhibited PTP1B (IC_50_ = 29.8 μg/mL), an effect not observed with PIC alone. Both samples reduced ROS production in human neutrophils, with PIC displaying high potency in the luminol assay (IC_50_ = 0.12 μg/mL). In zebrafish embryos, PESE was less embryotoxic than PIC, with LC_50_ values of 39.3 and 6.2 μg/mL, respectively. In the hyperglycemic zebrafish model, both samples significantly reduced total free glucose and oxidant activity (*p* ≤ 0.05) and induced distinct antioxidant gene-expression responses: PESE upregulated nfe2l2a, sod1, sod2, and cat, whereas PIC upregulated nfe2l2a, gpx1a, and cat. These findings demonstrate that PESE and PIC act through multiple complementary mechanisms to counteract hyperglycemia and oxidative stress, highlighting the therapeutic potential of an underutilized byproduct while emphasizing the importance of developmental toxicity assessment for the safe advancement of plant-derived agents.

## Graphical abstract

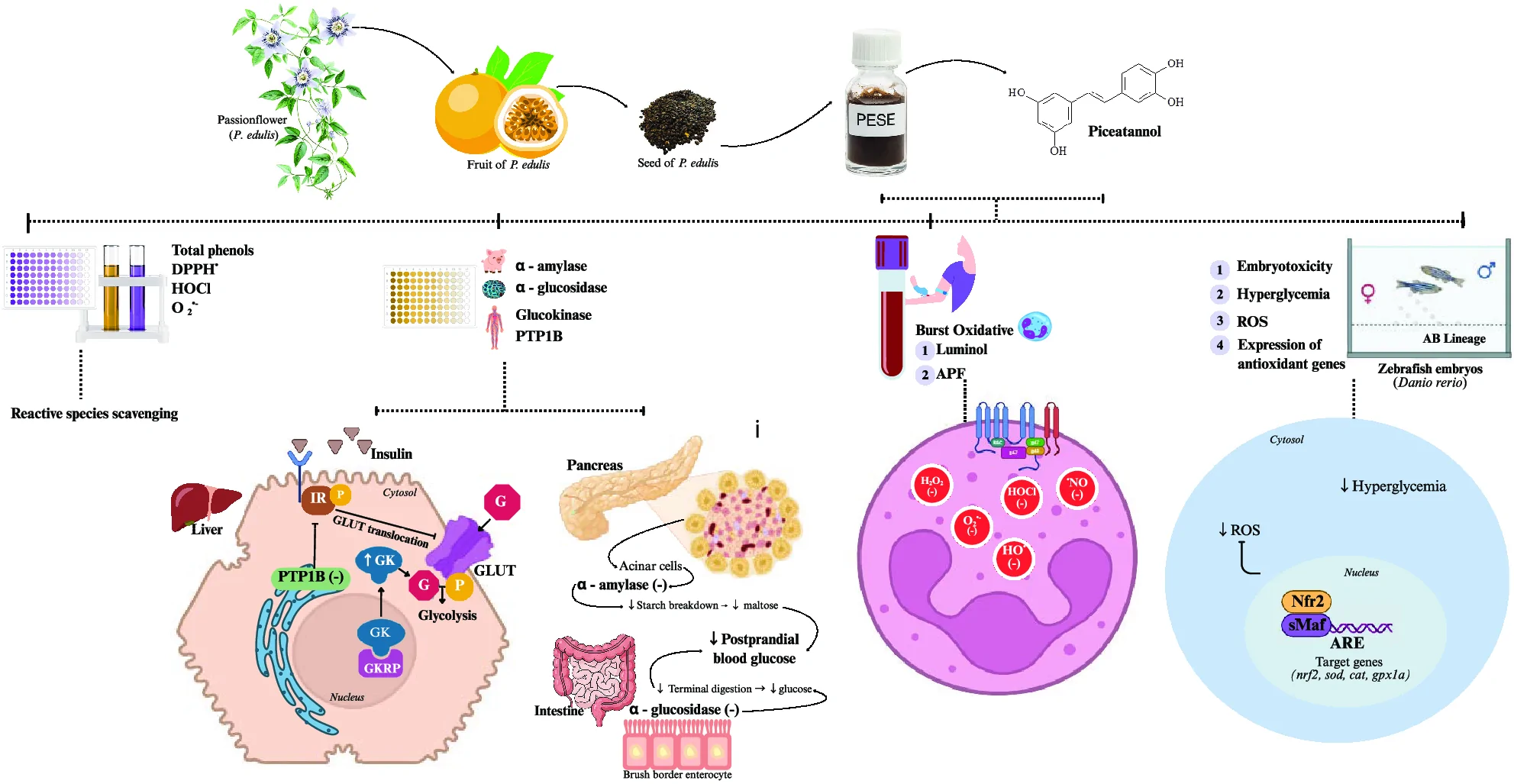

## 1 Introduction

Persistent oxidative stress has emerged as a central mechanism underlying metabolic dysregulation, particularly in conditions associated with insulin resistance.^1,2^ Characterized by excessive generation of reactive oxygen and nitrogen species (RONS) and impaired antioxidant defenses, oxidative stress compromises glucose utilization and perpetuates redox imbalance, establishing a vicious cycle that contributes to the progression of chronic diseases, especially diabetes mellitus (DM).^3,4^

In clinical practice, insulin resistance manifests in both type 2 diabetes mellitus (T2DM) and gestational diabetes mellitus (GDM).^5,6^ Although oral antidiabetic agents in T2DM and insulin therapy in GDM effectively control blood glucose levels, they do not directly interfere with oxidative stress pathways that sustain metabolic dysfunction.^7,8^ This limitation has driven growing interest in natural products as complementary therapeutic strategies capable of simultaneously modulating hyperglycemia and oxidative stress. In this context, agro-industrial byproducts emerge as sustainable and underexplored sources of bioactive phytochemicals, aligning therapeutic innovation with circular economy principles.^9,10^ Passion fruit (*Passiflora edulis*), widely cultivated in Brazil and other tropical regions, generates seeds that are often discarded as low-value byproducts despite being a rich source of piceatannol (PIC) and other bioactive phenolics. PIC, a catechol-substituted stilbene with recognized antioxidant, anti-inflammatory, and antidiabetic properties, remains underexplored and is often discarded as a low-value byproduct.^11–16^

Although the biological activities of *Passiflora edulis* seeds and PIC have been studied in several works, the present work offers a new contribution by performing the first direct comparative evaluation between the crude ethanolic seed extract (PESE) and isolated PIC through a comprehensive and multilevel approach. This study combines human and nonhuman antidiabetic enzymes (including glucokinase activation and PTP1B inhibition), human neutrophil oxidative burst assays, and an embryonic zebrafish hyperglycemia model with analysis of Nrf2-regulated antioxidant gene expression. Such an integrated strategy allows for a more robust assessment of the differential profiles, multitarget potential, and safety of the extract, in comparison to the pure compound, aspects still underexplored in the literature.

Therefore, this study aimed to perform a comparative experimental evaluation of PESE and isolated PIC, using complementary *in vitro* and embryonic *in vivo* models. Human and nonhuman antidiabetic enzymes, cellular assays including human neutrophil oxidative burst, and an embryonic zebrafish hyperglycemia model were employed to reveal key pathophysiological aspects of T2DM and GDM while adhering to the ethical principles of the 3Rs (Replacement, Reduction, and Refinement). Porcine pancreatic α-amylase and *Saccharomyces cerevisiae* α-glucosidase, two widely used nonhuman enzymes in preliminary antidiabetic screening, were included due to their high reproducibility, low cost, and well-established correlation with human enzymatic responses.^17^ Human enzymes (glucokinase and PTP1B) were used to provide more translational relevance to human physiology.^18–20^ Additionally, human neutrophil oxidative burst assays were performed to evaluate the modulation of cellular oxidative stress, while the embryonic zebrafish hyperglycemia model was employed as an integrated *in vivo* vertebrate system to assess systemic effects on glycemia, oxidative stress, and antioxidant gene expression.^21–23^

This study provides a unique contribution in the direct comparison of a chemically complex seed extract and isolated PIC across multiple and interconnected biological targets. This approach allows identifying activities preferentially associated with PIC and effects only observed for the whole extract, thus providing new evidence on their differential and potentially complementary antioxidant and antidiabetic profiles. Moreover, the present study contributes to the sustainable valorization of passion fruit seeds as an agro-industrial byproduct in a circular bioeconomy context.

## 2 Methods

### 2.1 Chemicals

All reagents were of analytical grade. Folin–Ciocalteu (FC), 2,2-diphenyl-1-picrylhydrazyl radical (DPPH^•^), β-nicotinamide adenine dinucleotide (β-NADH), *p*-nitroblue tetrazolium chloride (NBT), *N*-methylphenazonium methyl sulfate (PMS), sodium hypochlorite (NaOCl), dihydrorhodamine-123 (DHR-123), and 4-aminobenzylhydrazine (ABAH) were obtained from Sigma-Aldrich (Steinheim, Germany).

Porcine pancreatic α-amylase, *Saccharomyces cerevisiae* α-glucosidase (α-glucosidase), acarbose (ACB), 2-chloro-4-nitrophenyl-α-*D*-maltotrioside (CNPG3), 4-nitrophenyl-β-*D*-glucuronide (PNPG), and bovine serum albumin (BSA) were obtained from Sigma-Aldrich (Oakville, ON, Canada).

Protein tyrosine phosphatase 1B (PTP1B), *p*-nitrophenyl phosphate (pNPP), citric acid, dithiothreitol (DTT), glucokinase (GK), dorzagliatin, adenosine triphosphate (ATP), *D*-glucose, histopaque (densities 1.1191 g/mL and 1.0771 g/mL), NOX III inhibitor (3-benzyl-7-(2-benzoxazolyl)­thio-1,2,3-triazolo­[4,5-*d*]­pyrimidine, MM 360.39, VAS2870), calcium chloride (CaCl_2_), Trizma base, magnesium sulfate (MgSO_4_), Dulbecco’s phosphate-buffered saline (PBS) free of CaCl_2_ and magnesium chloride (MgCl_2_), phorbol-12-myristate-13-acetate (PMA), 0.4% trypan blue, and luminol were obtained from Sigma Chemical Co. (St. Louis, MO, USA).

Sodium chloride (NaCl) and potassium chloride (KCl) were obtained from VWR Chemicals (Alfragide, Portugal) and Pronalab (Abrunheira, Portugal), respectively. 2-[6-(4′-Amino)­phenoxy-3H-xanthen-3-on-9-yl]­benzoic acid (APF) was obtained from Invitrogen, Life Technologies Ltd. (Paisley, UK), while PIC (CAS: 10083-24-6) was obtained from Ambeed, Inc. (Illinois, USA). Methanol, dimethyl sulfoxide (DMSO), and hexane were obtained from Supelco (Supelco Park, PA, USA).

### 2.2 Plant Sample Acquisition and Preparation

*P. edulis* fruits were obtained from the organic passion fruit production of Fazenda Paraíso Agrícola, a family producer’s association in Matriz do Camaragibe, Alagoas, Brazil (9°09′24″S, 35°31′45″W). Access to Brazilian genetic heritage was registered in the National System for the Management of Genetic Heritage and Associated Traditional Knowledge (SisGen) under code A1CA908. The seeds were manually separated from the pulp, washed with running water, and oven-dried at 50 °C for 48 h until a constant weight was obtained.

Subsequently, the dried seeds were ground in an analytical mill (A11 Basic, IKA, Staufen, Germany) until a homogeneous flour was obtained. The lipid fraction was removed by Soxhlet extraction with hexane for 6 h. The defatted biomass was then extracted with absolute ethanol (p.a.) in a Soxhlet apparatus for an additional 6 h, using a continuous method. The resulting ethanolic extract was concentrated in a rotary evaporator (Büchi) under reduced pressure at 40 °C until complete removal of the solvent. The residue was further dried to constant weight, yielding PESE at 12.3 ± 1.5% (w/w) relative to the defatted seed powder. The dried extract was stored in amber glass bottles at 4 °C until use. For the biological assays, stock solutions were prepared by dissolving the dried PESE in DMSO or in an appropriate buffer, followed by serial dilutions to the desired concentrations.

### 2.3 Sample Characterization by Mass Spectrometry (LC-MS/MS)

#### 2.3.1 LC-HRMS Instrumentation

The operating conditions of the mass spectrometry system (Orbitrap QE, Thermo Scientific, Bremen, Germany) were configured as follows: resolution of 70,000, 1 microscan, AGC target of 1 × 10^6^, and maximum injection time of 100 ms. Gas parameters were set with sheath gas at 10 units, auxiliary gas at 5 units, and sweep gas at 0 units. Spray voltage was adjusted to 3.1 kV in negative mode and 3.9 kV in positive mode. Capillary temperature was maintained at 320 °C, and the S-lens RF level was set to 45. A data-dependent MS/MS (ddMS2) method was used with a ddMS2 resolution of 35,000 and an AGC target of 1 × 10^5^, fragmenting the 10 most intense ions (TopN) using collision energies of 20, 25, and 30 eV in the collision cell.

The liquid chromatograph used was a high-performance liquid chromatography (HPLC) Agilent 1260 Infinity II (Agilent Technologies, Santa Clara, California). The chromatographic method was based on the method of Sie et al.^24^ The column used was a ZORBAX EC-C18 (4.6 × 150 mm, 5 μm), with the eluting solvent being a mixture of trifluoroacetic acid and acetonitrile in a fixed ratio of 75:25, at a continuous flow rate of 5 mL/min.

#### 2.3.2 Analytical Procedure and Metabolite Identification

The chemical composition of the ethanolic extract (PESE) was characterized using liquid chromatography coupled to high-resolution mass spectrometry (LC-HRMS), employing both positive and negative electrospray ionization (ESI) modes to maximize metabolite coverage.

Metabolite identification was performed by comparing high-resolution MS/MS fragmentation spectra with previously reported data and bibliographic references. Mass accuracy values (1.7 ppm for PIC, 1.2 ppm for scirpusin B, and 2.4 ppm for naringenin) were obtained using Xcalibur software (Thermo Fisher Scientific, Waltham, MA, USA), and values <5 ppm were considered acceptable for structural confirmation.

### 2.4 Antioxidant Activity and Phenolic Content Assay

#### 2.4.1 Determination of Total Phenol Content (TPC)

TPC was determined in PESE using the FC method. For sample analysis, the PESE was tested at a fixed concentration of 50 μg/mL. Subsequently, 300 μL of FC reagent, 2.4 mL of 5% (w/v) sodium carbonate (Na_2_CO_3_), and 180 μL of deionized water were added to the centrifuge tubes.^25^ The mixture was shaken for 18 min and incubated in a 40 °C water bath, in the dark, for 20 min. After cooling to room temperature, absorbance was measured at 700 nm using a UV–vis spectrophotometer (Agilent 8453). Quantification was performed using a gallic acid (GA) calibration curve, and the results were expressed as mg gallic acid equivalents per gram of dry extract (mg GAE/g). A minimum of three independent assays were performed, each with triplicate samples.

#### 2.4.2 DPPH^•^ Scavenging Assay

The antioxidant capacity was determined for both PESE (0.78–50 μg/mL) and PIC (0.39–48 μg/mL),^26^ with minor modifications. A methanolic solution of DPPH^•^ (40 μg/mL, HPLC grade, 98%) was prepared, and 2.7 mL of this solution was added to 0.3 mL of PESE (0.78–50 μg/mL), PIC (0.39–48 μg/mL/1.60–196.5 μM), or quercetin (0.4–15 μg/mL/1.32–49.6 μM) at the tested concentrations. All experiments were conducted at least three times independently, with measurements performed in triplicate. The mixture was incubated at room temperature in the dark for 30 min, after which the decrease in absorbance at 516 nm was measured spectrophotometrically. A curve of inhibition (%) versus concentration (μg/mL) was constructed to calculate the IC_50_.

#### 2.4.3 HOCl Scavenging Assay

The scavenging effect of PESE and PIC against HOCl was evaluated by fluorescence.^27^ HOCl solution was prepared by adjusting the pH of a 1% (w/v) NaCl solution to 6.2 with the dropwise addition of 10% H_2_SO_4_ and then diluting it to 30 μM in 100 mM phosphate buffer (pH 7.4). In a 96-well plate, 150 μL of buffer, 50 μL of PESE (0.78–50 μg/mL) or PIC (0.39–25 μg/mL/1.6–102.4 μM) or quercetin (0.4–60 μg/mL/1.32–198.6 μM), 50 μL of DHR-123 (5 μM), and 50 μL of HOCl (5 μM) were added. Fluorescence was measured at 37 °C (λ_excitation_ = 505 ± 10 nm; λ_emission_ = 530 ± 10 nm) using a microplate reader (Infinite 200 PRO). Results were expressed as IC_50_ (μg/mL). Each assay consisted of at least three independent experiments (*n* ≥ 3), performed in triplicate.

#### 2.4.4 Superoxide Radical Anion Scavenging Capacity (O_2_^•–^)

The O_2_^•^ scavenging potential was evaluated,^27^ with adaptations. In a 96-well plate, 50 μL of PESE (0.78–50 μg/mL) or PIC (0.39–25 μg/mL/1.6–102.4 μM) or quercetin (4–60 μg/mL/13.2–198.6 μM), 50 μL of NADH (166 μM), 150 μL of NBT (43.3 μM), and 50 μL of PMS (2.7 μM) were added, all dissolved in 19 mM phosphate buffer (pH 7.4). Data were obtained from at least three independent experiments, each conducted in triplicate. The assay was performed at 37 °C using a microplate reader (Infinite 200 PRO), and absorbance was measured at 560 nm. Results were expressed as IC_50_ (μg/mL).

### 2.5 In Vitro Antidiabetic Activity Assays

#### 2.5.1 Assessment of GK Enzyme Activity

GK activity was assessed using a commercial Glucokinase Assay Kit (MAK441; Merck, Darmstadt, Germany), according to the manufacturer’s instructions. Briefly, 25 μL of the enzyme suspension (1.25 μg/mL) and 5 μL of PESE (2.5–40 μg/mL) or PIC (0.15–2.5 μg/mL/0.61–10.2 μM) were incubated for 15 min at 37 °C. Subsequently, ATP (100 μM) and d-glucose (10 mM) were added, and the reaction was allowed to proceed at 37 °C. After this step, the working reagent supplied with the kit was added. The reaction was monitored fluorometrically in a microplate reader (Synergy HT, BIOTEK) at 37 °C using filter-based detection, with excitation at 530 nm (bandpass 530 ± 25 nm) and emission at 590 nm (bandpass 590 ± 35 nm). Dorzagliatin was used as the positive control. Experiments were performed at least three times independently, with each experiment having triplicate determinations.

#### 2.5.2 PTP1B Activity Inhibition Assay

The PTP1B inhibition assay was performed with some modifications.^19^ Briefly, a suspension of the human PTP1B enzyme (2 μg/mL) in 50 mM citrate buffer containing 50 mM citric acid, 0.1 M NaCl, 1 mM EDTA and 1 mM DTT (pH 6.0) was preincubated for 2 min at 37 °C with PESE (2.5–40 μg/mL) or PIC (0.15–2.5 μg/mL/0.61–10.2 μM), dissolved in 1% DMSO, using a 96-well plate. Subsequently, pNPP (2 mM) in citrate buffer was added to initiate the catalytic reaction. The reaction mixture was monitored spectrophotometrically in a microplate reader (Synergy HT, BIOTEK) at 405 nm for 30 min at 37 °C. Ursolic acid (9–22 μg/mL/19.7–48.2 μM) was used as the positive control. The obtained values correspond to the slope of the reaction kinetics, as determined from the absorbance increments between 2 and 10 min. All studies were conducted in triplicate across three or more independent experiments.

#### 2.5.3 α-Amylase Inhibition Assay

All solutions were prepared in PBS (0.01 M) containing NaCl (60 mM) and sodium azide (0.02% w/v), with a pH of 6.8. In a 96-well plate, 20 μL of PESE (0.08–80.0 μg/mL) or PIC (0.02–6.0 μg/mL/0.082–24.6 μM), 20 μL of buffer, and 20 μL of porcine pancreatic α-amylase (4 U/mL) were added.^28^ After incubation for 10 min at 37 °C, 20 μL of CNPG3 (5 mM) was added, and the mixture was incubated for an additional 30 min at 37 °C. The reaction was stopped with 240 μL of trisodium phosphate (1% w/v, pH 11). Absorbance at 405 nm was measured in a microplate reader (Infinite 200 PRO, TECAN). Acarbose (0.1–10.0 μg/mL/0.15–15.5 μM), an α-amylase inhibitor used in the treatment of T2DM, was used as the positive control. For each study, at least three independent experiments were performed, each conducted in triplicate.

#### 2.5.4 α-Glucosidase Inhibition Assay

All solutions were prepared in 0.01 M potassium phosphate buffer, pH 6.8. In 96-well plates, 120 μL of PESE (0.8–80.0 μg/mL) or PIC (0.02–6.0 μg/mL/0.082–24.6 μM) was added together with 20 μL of α-glucosidase (0.35 U/mL).^29^ The mixture was incubated at 37 °C for 15 min, followed by the addition of 20 μL of the substrate 4-nitrophenyl-β-d-glucuronide (5 mM). After an additional 15 min incubation at 37 °C, the reaction was stopped with 80 μL of sodium carbonate (0.2 M, prepared in 0.1 M potassium phosphate buffer, pH 6.8). Absorbance was measured at 405 nm using a microplate reader (Infinite 200 PRO, TECAN) to quantify p-nitrophenol release. Acarbose was used as the positive control at concentrations of 15–500 μg/mL (23.2–774.4 μM).

### 2.6 Assessment of the Oxidative Burst in Human Neutrophils

All donor-related procedures and protocols were conducted in accordance with the Declaration of Helsinki and were approved by the Ethics Committee of the Porto Hospital Centre, Portugal. After written informed consent was obtained, venous blood was collected from healthy human donors by antecubital venipuncture into K_3_EDTA vacuum tubes. Human neutrophils were isolated by density gradient centrifugation.^30^

The study used PMA to stimulate human neutrophils and assess their reactive species-generating capacity. For this purpose, chemiluminescence and fluorescence methods using luminol and APF as probes, respectively, were adapted for use in a microplate reader (Synergy HT, BIO-TEK), as detailed below. Each study comprised at least five independent experiments, performed in triplicate.

#### 2.6.1 Isolation of Human Neutrophils

Human neutrophils were isolated by the density gradient centrifugation method^30^ with minor modifications. Briefly, in a 15 mL polypropylene tube, the following reagents were added in sequence: histopaque 1.119 g/mL (3 mL), histopaque 1.077 g/mL (3 mL), and the collected blood (4.5 mL). The samples were then centrifuged at 900*g* for 30 min at 20 °C. After centrifugation, the neutrophil-containing layer was collected and washed with PBS. The suspension was centrifuged at 850*g* for 5 min at 4 °C. The supernatant was discarded, and the neutrophil pellet was resuspended in PBS; Milli-Q water was then added, followed a few minutes later by 3% (w/v) NaCl to restore isotonicity. The suspension was centrifuged again at 850*g* for 5 min at 4 °C.

The supernatant was discarded, yielding a purified neutrophil pellet. After isolation, the pellet was resuspended in Tris–glucose buffer (25 mM Trizma base, 5.5 mM d-glucose, 140 mM NaCl, 1.26 mM CaCl_2_, 0.81 mM MgSO_4_, 5.37 mM KCl, pH 7.4). The neutrophil suspension was kept on ice under gentle agitation until further use. Cell yield and viability were determined by the trypan blue exclusion method.

#### 2.6.2 Assessment of Neutrophils’ Oxidative Burst

##### 2.6.2.1 Luminol Oxidation

The chemiluminescent probe luminol has been extensively used to monitor the production of reactive species by neutrophils, namely, the O_2_^•–^, hydrogen peroxide (H_2_O_2_), hydroxy radical (HO^•^), hypochlorous acid (HOCl), nitric oxide (^•^NO), and peroxynitrite anion (ONOO^–^).^22^ The measurement of neutrophils’ oxidative burst was performed by monitoring the oxidation of luminol.^21^ In a 96-well microplate, human neutrophils (1 × 10^6^ cells/mL) were incubated with luminol (500 μM) and either PESE (0.62–10 μg/mL) or PIC (0.07–2.5 μg/mL/0.29–10.24 μM) for 5 min at 37 °C under continuous gentle shaking before stimulation with PMA (160 nM). Kinetic readings were initiated immediately after cell stimulation, and measurements were recorded at the peak response, which typically occurred approximately 15 min later. Results are expressed as the percentage inhibition of luminol oxidation, with VAS2870 (0.11–3.6 μg/mL/0.30–9.99 μM) serving as the positive control. All experiments were performed more than five times, with each repetition carried out in triplicate.

##### 2.6.2.2 APF Oxidation

APF is a nonfluorescent fluorescein derivative that is oxidized by HOCl, ONOO^–^, and HO^•^, becoming a fluorescent probe. Its selectivity was tested using the myeloperoxidase (MPO) inhibitor ABAH. The addition of ABAH to human neutrophils stimulated with PMA, decreased the APF-dependent fluorescence signal to the level of the control assay, ruling out the involvement of HOCl, HO^•^ and ONOO^–^.^22^ The measurement of the neutrophils’ oxidative burst was performed by fluorescence, monitoring the oxidation of APF induced by HOCl.^30,31^ In 96-well microplates, human neutrophils (1 × 10^6^ cells/mL) were incubated with APF (1 μM) and the test compounds [PESE (2.5–40 μg/mL), PIC (0.035–1.2 μg/mL/0.14–4.91 μM)] for 5 min at 37 °C under continuous gentle stirring, followed by stimulation with PMA (160 nM). Kinetic readings were initiated immediately thereafter using excitation and emission wavelengths of 500 and 520 nm, respectively. Data were calculated from the slope of the fluorescence curves between 15 and 30 min and expressed as the percentage inhibition of APF oxidation. ABAH (1.7–27.4 μg/mL/11.2–181.2 μM) was included as the positive control. Data represent over five independent repetitions, each conducted in triplicate.

### 2.7 Animal Model and Management

This experimental preclinical study was approved by the Animal Use Ethics Committee of the Federal University of Alagoas (UFAL) under protocol no. 13/2022. Acute toxicity was assessed using the Fish Embryo Acute Toxicity (FET) test, conducted according to OECD guideline no. 236,^32^ to determine the median lethal concentration (LC_50_). Sublethal concentrations were subsequently defined based on standard toxicological parameters, including the no observed adverse effect level (NOAEL) and LC_50_, following established principles for preclinical studies with therapeutic potential.^33^

Zebrafish (*Danio rerio*) embryos (AB strain) were used as the biological model. Embryos were obtained by natural spawning of adult breeders maintained under controlled laboratory conditions. Fertilized embryos were collected within 1 h postfertilization (hpf), examined under a stereomicroscope to confirm viability, and only batches presenting fertilization rates ≥ 70% and coagulation rates <10% in the negative control were included in the experiments.

Adult fish were maintained in a recirculating aquaculture system under controlled temperature, pH, dissolved oxygen, and photoperiod conditions, following standard husbandry procedures. Spawning was induced by light onset, and embryos at early developmental stages were selected for experimental exposure.

#### 2.7.1 Sample Preparation and Experimental Design

The concentrations of PESE were evaluated at 6.2–100 μg/mL, and of PIC at 0.6–19 μg/mL (2.5–77.8 μM). The substances were initially dissolved in 0.1% DMSO and diluted in system water to a final volume of 100 mL. Fertilized embryos were washed and individually distributed in 24-well polystyrene plates (1 embryo per well). Each experimental group consisted of 20 embryos, with an additional 4 embryos used as internal controls per plate. The assays were performed in duplicate, totalling 40 embryos per group, which included a positive control (3,4-dichloroaniline at 4 mg/L/24.7 μM), a negative control (system water), a solvent control (0.1% DMSO), PESE, and PIC.

#### 2.7.2 Determination of LC_50_ and Evaluation of Sublethal Effects in Danio rerio Embryos

To assess acute toxicity and determine the LC_50_ (Supporting Table 1), *Danio rerio* embryos were exposed to different concentrations of the compounds for 96 h. Twenty viable embryos per concentration (1 embryo/well) were used, with exposure starting as early as possible after fertilization and before blastodisc cleavage. To ensure randomness and viability, excess fertilized eggs were collected and transferred to Petri dishes containing 100 mL of the test solutions, respecting the maximum limit of 90 min postfertilization. Up to 180 min postfertilization, viable embryos were isolated, washed, and distributed into 24-well plates containing 2 mL/well of solutions prepared immediately before use.

A semistatic exposure model was adopted, with complete renewal of the solutions every 24 hpf to minimize compound degradation, maintain stable environmental conditions (photoperiod, oxygenation, and temperature), and remove metabolites or toxic residues that could interfere with the biological response.

Throughout the 96 hpf period, daily evaluations were performed under a stereomicroscope, focusing on apical parameters and embryonic development. Lethality criteria included embryonic coagulation, absence of somites, failure of tail detachment, and absence of heartbeat, with the presence of any of these features indicating death.

Sublethal effects were also monitored, including eye development, spontaneous movements, heart rate, pigmentation, edema, and anatomical malformations (head, heart, tail, dorsal cord, and otolith structures). Hatching was recorded from 48 hpf onward, with daily monitoring until the end of the trial.

Based on the cumulative mortality, a dose–response curve was constructed to calculate the LC_50_ of each substance. From these values, standard sublethal concentrations (1/10 and 1/20 of the LC_50_) were defined for subsequent studies of nonlethal effects with therapeutic potential.

#### 2.7.3 High Glucose Exposure and Quantification of Total Free Glucose

To induce hyperglycaemia and quantify total free glucose, *Danio rerio* embryos (*n* = 60 per group) were exposed to d-glucose (2% w/v) in 6-well plates containing 10 mL per well. Exposure started at 3 hpf and continued until 96 hpf.^23^ Experimental groups received the tested treatments simultaneously with glucose. The negative control was maintained in system water, whereas the positive control received insulin (0.1 U/mL) for 2 h to assess glucoregulatory efficacy.

Two exposure regimes were employed: (i) a pulsatile approach, consisting of alternating periods of treatment and recovery in system water at 48, 72, and 96 hpf; and (ii) continuous exposure, with complete renewal of solutions every 24 h.

At 96 hpf, embryos were collected, washed three times, and euthanised by cooling. Each sample (60 embryos) was transferred to sterile 2 mL tubes, residual vehicle was removed, and cell lysis was performed by sonication in 200 μL PBS (FisherBrand FB120, 50% amplitude, 10 s). The homogenate was centrifuged at 14,000 rpm (20,800*g*) for 2 min, and the supernatant collected.

Total free glucose was quantified using a portable glucometer (Accu-Chek Active, Roche). A volume of 1.5 μL of supernatant was applied per reading, and three consecutive measurements were obtained for each sample. Two independent biological replicates were performed to ensure analytical robustness.

#### 2.7.4 Reactive Oxygen Species (ROS) Analysis

ROS quantification was performed in *Danio rerio* larvae at 96 hpf. Twenty larvae per group were selected and euthanised by immersion in ice. The specimens were then washed three times with ice-cold PBS (pH 7.4) to remove extracellular residues that could interfere with subsequent analyses.^34^

The larvae were then subjected to cell lysis by sonication in 200 μL of PBS using a sonicator (FisherBrand, model FB120; Thermo Fisher Scientific, CA, USA) operating at 50% amplitude for 10 s. The homogenates were centrifuged at 10,000*g* for 10 min at 4 °C, and the supernatants were collected for oxidative activity analysis.

Oxidative activity in the homogenates was detected using the fluorescent probe 2′,7′-dichlorodihydrofluorescein diacetate (H_2_DCFDA, Thermo Fisher Scientific, CA, USA). Because H_2_DCFDA activation requires intracellular esterases, in this experimental design the resulting signal reflects overall oxidant activity in the homogenates rather than exclusively intracellular ROS. For the reaction, 40 μL of each supernatant sample was incubated with 40 μM of the probe in the dark at 37 °C for 30 min.

Fluorescence readings were performed using a multimodal microplate reader (GloMax Multi Detection System; Promega, Madison, WI, USA) with excitation and emission wavelengths of 490 and 530 nm, respectively. Each condition was analyzed in three independent biological replicates, with each replicate measured in technical quintuplicate.

ROS levels were expressed as a percentage relative to the negative control group, enabling quantitative assessment of oxidative stress induced by the different treatments.

#### 2.7.5 RNA Extraction and Quantitative Real-Time Polymerase Chain Reaction (qPCR)

At 96 hpf, 20 *Danio rerio* larvae from each experimental group were euthanized by cooling and immediately preserved in RNAlater stabilizing solution (Thermo Fisher Scientific, USA) to maintain the integrity of the total RNA. RNA extraction and purification were performed using the PuriLink RNA Mini kit (Life Technologies, Carlsbad, CA, USA), according to the manufacturer’s instructions. The concentration and purity of the extracted RNA were assessed by spectrophotometry using the NanoDrop One microvolume UV–vis spectrophotometer (Thermo Fisher Scientific, Waltham, MA, USA).

All RNA samples were standardized to a final concentration of 100 ng/μL before cDNA synthesis. Reverse transcription was performed using the High-Capacity cDNA Reverse Transcription Kit (Applied Biosystems, USA) following the manufacturer’s protocol. For qPCR, 10 ng of cDNA per reaction was used in a total reaction volume of 10 μL (5 μL SYBR Green Master Mix, 1 μL forward primer, 1 μL reverse primer, 2 μL nuclease-free water, and 1 μL cDNA).

qPCR reactions were performed with the SYBR Green qPCR kit (Qiagen, CA, USA) on a Rotor-Gene Q system (Qiagen, CA, USA). Relative gene expression analysis was performed using the 2^^−ΔΔCt^ comparative method. The transcription levels of six genes related to oxidative stress were quantified: *superoxide dismutase 1 (sod1), superoxide dismutase 2 (sod2), catalase (cat), glutathione peroxidase 1a (gpx1a), glutathione reductase (gsr), and nuclear factor erythroid 2–related factor 2 (nfe2l2a)* ^35^. The reference gene used was β*-actin* (*actb*),^36^ previously validated for its stability under experimental treatments, compared with the candidate genes *elfa* (elongation factor 1-α) and *gapdh* (glyceraldehyde-3-phosphate dehydrogenase*)*.

All assays were performed in technical triplicate, and the procedures strictly followed the guidelines established by Minimum Information for Publication of Quantitative Real-Time PCR Experiments (MIQE), ensuring the reproducibility and reliability of the data obtained.^37,38^ Supporting Table 2 presents the accession numbers of the gene sequences, the sequences of the primers specific to each gene, the expected amplicon size (bp) and the dissociation temperature (Tm) used.

### 2.8 Statistical Analysis

All statistical analyses were performed using GraphPad Prism, version 9.0 (GraphPad Software, San Diego, CA, USA). Data are presented as mean ± standard error of the mean (SEM), and differences were considered statistically significant at *p* ≤ 0.05. For comparison of antioxidant activity (DPPH^•^, O_2_^•–^, and HOCl scavenging assays) and antidiabetic enzymatic assays, data on the inhibition of α-amylase, α-glucosidase, and GK were analyzed using one-way analysis of variance (ANOVA) followed by Tukey’s post hoc test. In contrast, PTP1B inhibition was assessed using the unpaired Student’s *t* test, due to the comparison of two experimental groups. The oxidative burst assay in human neutrophils, using luminol and APF probes, was analyzed by one-way ANOVA followed by Tukey’s post hoc test.

For the embryotoxicity assays in Danio rerio, LC_50_ values were calculated using nonlinear regression analysis (log­[inhibitor] vs normalized response with variable slope), without applying additional inferential statistical tests. Developmental abnormalities were summarized using heatmaps representing the frequency of observed outcomes on a color scale ranging from 0 (white) to 5 (red), without statistical comparison. Embryo survival rates under sublethal exposure (1/10 and 1/20 of the LC_50_) were presented descriptively, as this analysis aimed to identify response trends at low doses and provide complementary insights to the primary toxicological evaluation.

To assess the antidiabetic effect under pulsatile hyperglycaemia in Danio rerio, data were analyzed using one-way ANOVA followed by Dunnett’s post hoc test, comparing the treatment groups with the hyperglycaemic control. In the continuous hyperglycaemia model, data were subjected to a two-way ANOVA, followed by Dunnett’s post hoc test for comparisons with the hyperglycaemic control group and Tukey’s post hoc test for comparisons with the insulin-treated group. This approach enabled assessment of: (i) the interaction between treatment and exposure time; (ii) the ability of the tested compounds to reverse glucose-induced hyperglycaemia; and (iii) whether their effect was comparable to or greater than that of insulin. Finally, the analysis of ROS production and relative gene expression related to oxidative stress in *Danio rerio* larvae exposed to continuous hyperglycaemia was conducted using one-way ANOVA followed by Tukey’s post hoc test.

## 3 Results

### 3.1 Extraction and Chemical Profiling of PESE

The moisture content of *P. edulis* dry seeds was 6.92% at 105 °C. Ethanolic extraction yielded PESE at 12.3 ± 1.5%. The chemical profile of PESE was characterized by LC-HRMS using both positive and negative ESI modes. Although metabolites were detected in both ionization conditions, three major phenolic compounds—PIC (*m*/*z* 243.071), scirpusin B (*m*/*z* 485.121) and naringenin (*m*/*z* 271.061)—were confidently identified in negative ESI mode based on accurate mass measurements and MS/MS fragmentation patterns.

Metabolite identification was supported by comparison with reported MS/MS data, with mass errors of 1.7 ppm for PIC, 1.2 ppm for scirpusin B, and 2.4 ppm for naringenin, all within the accepted threshold (<5 ppm) for high-confidence molecular assignment and processed using Xcalibur software (Thermo Fisher Scientific, Waltham, MA, USA).

PIC fragmentation showed characteristic neutral losses of water (−18 Da) and ketene (−42 Da), consistent with hydroxylated stilbene structures.^39^ Scirpusin B exhibited fragmentation patterns indicative of dimer cleavage, generating stilbene-type ions near *m*/*z* 243.071 confirming its dimeric nature.^24^ Naringenin displayed typical Retro-Diels–Alder fragmentation of the C-ring, yielding diagnostic ions at *m*/*z* 151 and 119 ^40^ (Supporting Figures 1–3).

The predominance of negative ionization is consistent with the acidic phenolic nature of these compounds, which favors [M–H]^−^ formation and enhances sensitivity for flavonoids and stilbenoids.^41^ Together, these data confirm the reliable identification of the major phenolic constituents of PESE (Table 1).

**Table 1:**
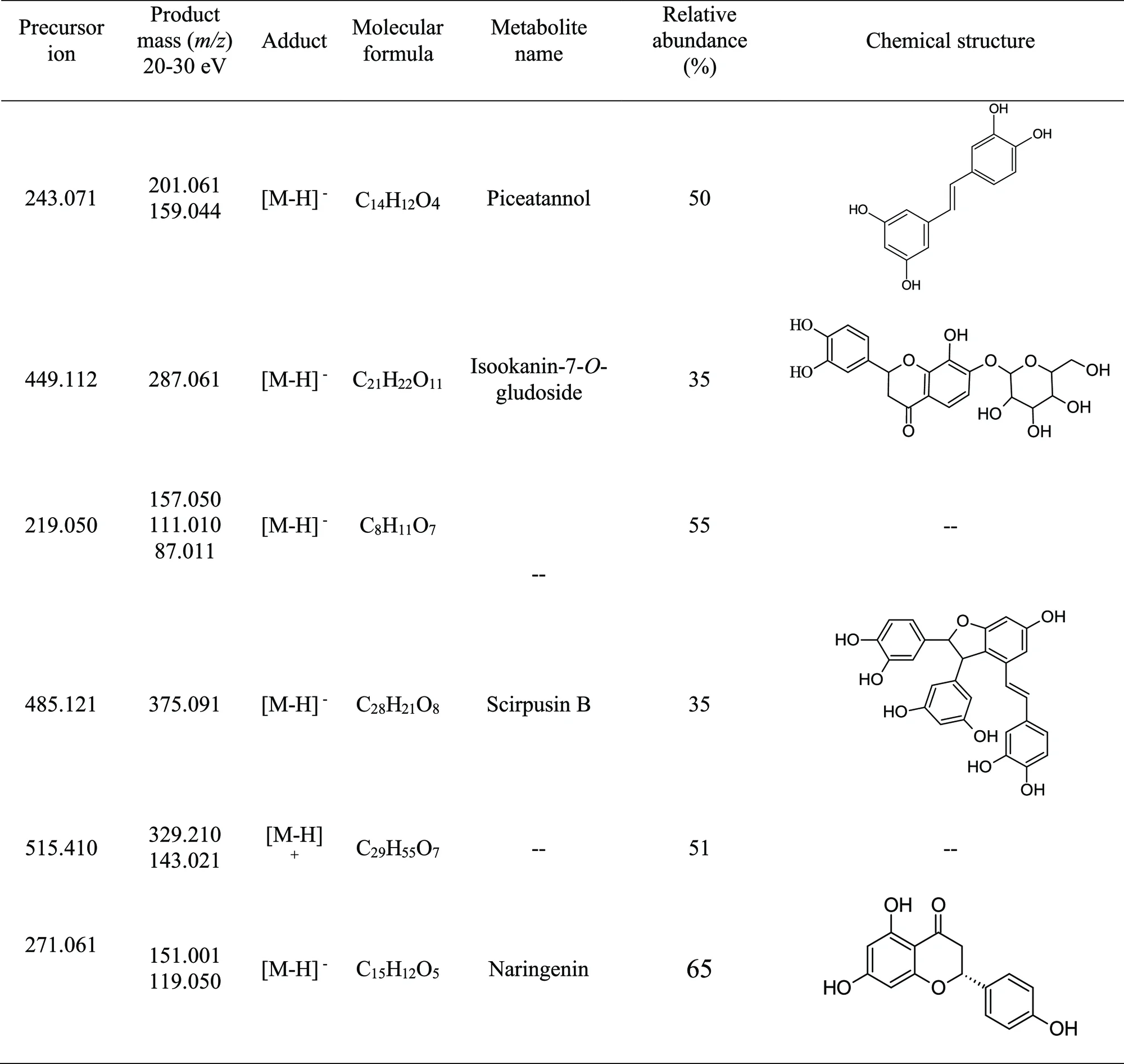
Identification of Metabolites in *P. edulis* Seed Ethanol Extract by Mass Spectrometry^a^

a *m*/*z*: Mass-to-charge ratio; eV: applied collision energy.

### 3.2 TPC and Antioxidant Capacity

The TPC and antioxidant activities of PESE, isolated PIC, and reference compound quercetin are presented in Supporting Table 4. PESE exhibited a TPC of 238.9 ± 1.9 mg GAE/g dry extract. In chemical assays evaluating scavenging capacity against DPPH^•^, O_2_^•–^, and a nonradical oxidant species (HOCl), both PESE and PIC exhibited significant antioxidant activity across all systems assessed (*p* ≤ 0.05).

PIC consistently showed greater potency than PESE, as indicated by lower IC_50_ values, approximately 3.7× in DPPH^•^ (3.9 vs 14.5 μg/mL), 10.7× in HOCl (0.5 vs 5.4 μg/mL), and 1.7× in O_2_^•–^ scavenging (8.3 vs 13.9 μg/mL). Notably, both samples performed best against HOCl.

Quercetin exhibited the highest antioxidant activity in the DPPH^•^ assay (1.4 μg/mL; 4.7 μM), outperforming both PESE and PIC. In the HOCl assay (1.3 μg/mL; 4.4 μM), its activity was comparable to PIC. Conversely, in the O_2_^•–^ system, PESE and PIC showed significantly higher scavenging capacity than quercetin (*p* ≤ 0.05). These results demonstrate that, while quercetin is a potent general antioxidant, PESE and PIC display selective and enhanced activity against specific reactive species, particularly O_2_^•–^ and HOCl.

### 3.3 Modulation of Glucose Metabolism Enzymes

The modulatory effects of PESE and PIC on enzymes of glucose metabolism are summarized in Table 2. PIC inhibited the activities of porcine α-amylase and *S. cerevisiae* α-glucosidase with IC_50_ values of 11.8 ± 0.5 μg/mL (48.2 μM) and 3.1 ± 0.1 μg/mL (12.6 μM), respectively, both significantly lower than those of PESE (47.9 ± 2.9 and 16.1 ± 1.0 μg/mL; *p* ≤ 0.05). Considering the positive controls, PIC was far less potent than acarbose against α-amylase (1.0 ± 0.1 μg/mL; 1.5 μM), but far more potent than acarbose against α-glucosidase (153.4 ± 3.1 μg/mL; 237.6 μM). In assays with the human enzyme PTP1B, PESE showed inhibition (IC_50_ = 29.8 ± 2.7 μg/mL), whereas PIC displayed no activity up to 2.5 μg/mL; the reference ursolic acid was more potent (IC_50_ = 16.3 ± 0.9 μg/mL; 35.7 μM). Both samples activated glucokinase (GK), with PIC markedly more efficacious than PESE (IC_50_: 0.4 ± 0.0 vs 11.6 ± 1.6 μg/mL), yet less potent than the positive control dorzagliatin (0.1 ± 0.0 μg/mL; 0.2 μM).

**Table 2:** Regulatory Effects on Glucose Metabolism and Cell Signaling of *P. edulis* Seed Ethanolic Extract and Piceatannol[Table-fn t2fn1]

	**IC**_**50**_ **(μg/mL)**			
**sample**	**α-amylase**	**α-glucosidase**	**PTP1B**	**GK**
**PESE**	47.9 ± 2.9^c^	16.1 ± 1.0^b^	29.8 ± 2.7^b^	11.6 ± 1.6^b^
**PIC**	11.8 ± 0.5^b^ (48.2 μM)	3.1 ± 0.1^a^ (12.6 μM)	inactive up to 2.5 μg/mL	0.4 ± 0.0^a^ (1.6 μM)
**positive control**				
acarbose	1.0 ± 0.1^a^ (1.5 μM)	153.4 ± 3.1^c^ (237.6 μM)		
ursolic acid			16.3 ± 0.9^a^ (35.7 μM)	
dorzagliatin				0.1 ± 0.0^a^ (0.2 μM)

* Mean ± SEM, *n* ≥ 3. α-Amylase, α-glucosidase, and GK were analyzed using one-way ANOVA followed by Tukey’s post hoc test, whereas PTP1B was analyzed using the unpaired Student’s *t*-test. Within each assay, values with different superscript letters are significantly different (*p* ≤ 0.05).

### 3.4 Inhibition of Human Neutrophils’ Oxidative Burst

According to our results, both PESE and PIC inhibited the PMA-induced oxidative burst in human neutrophils (Table 3). In the luminol assay, PIC showed the lowest IC_50_ (0.1 ± 0.0 μg/mL; 0.5 μM), markedly more potent than PESE (2.0 ± 0.1 μg/mL; *p* ≤ 0.05). PIC also displayed a lower IC_50_ than the NADPH oxidase inhibitor VAS2870 (0.5 ± 0.0 μg/mL; 1.3 μM). In the APF assay, PIC showed a markedly lower IC_50_ than PESE (0.4 ± 0.0 vs. 2.7 ± 1.2 μg/mL; ∼ 7-fold). Both samples were significantly more potent than ABAH [4.0 ± 0.4 μg/mL (26.8 μM); *p* ≤ 0.05].

**Table 3:** Inhibition of PMA-Induced Oxidative Burst in Human Neutrophils by *P. edulis* Seed Ethanolic Extract and Piceatannol, Using Luminol and APF Probes^a^

	**IC**_**50**_ **μg/mL (μM)**	
**sample**	**luminol**	**APF**
**PESE**	2.0 ± 0.1^b^	2.7 ± 1.2^b^
**PIC**	0.1 ± 0.0^a^ (0.5 μM)	0.4 ± 0.0^a^ (1.6 μM)
**positive control**		
VAS2870	0.5 ± 0.0^a^ (1.3 μM)	0.2 ± 0.0^a^ (0.4 μM)
ABAH		4.0 ± 0.4^c^ (26.8 μM)

a Mean ± SEM, *n* ≥ 5. Superscripts of different letters within the same assay represent significant differences (ANOVA followed by Tukey’s test, *p* ≤ 0.05). IC_50_: half-maximum inhibitory concentration; PESE: *P. edulis* seed dry ethanolic extract; PIC: Piceatannol; VAS2870: NOx III inhibitor (3-benzyl-7-(2-benzoxazolyl)­thio-1,2,3-triazolo­[4,5-*d*]­pyrimidine); ABAH: 4-aminobenzylhydrazine. IC_50_ values are expressed in μg/mL, with approximate molar equivalents (μM) calculated according to molecular weights: PIC (244.24 g/mol), VAS2870 (360.39 g/mol), and ABAH (151.17 g/mol)

### 3.5 Embryotoxicity and Sublethal Effects of PESE and PIC in Danio rerio

Physicochemical parameters of the embryo medium remained stable during exposure to different concentrations of PESE and PIC, with temperatures ranging from 26.0 to 26.2 °C and pH values between 6.7 and 7.2 (Supporting Table 3), confirming the controlled experimental conditions. The concentration–response analysis revealed distinct embryotoxicity profiles for the tested compounds (Supporting Figure 4). For PESE, the LC_50_ was estimated at 39.3 μg/mL (logLC_50_ = 1.6), whereas PIC exhibited a markedly lower LC_50_ of 6.2 μg/mL (25.4 μM) (logLC_50_ = 0.8). These data indicate that, under the experimental conditions, PIC exerts higher embryotoxicity than PESE.

Embryo survival was assessed over 144 hpf following exposure to sublethal concentrations (1/10 and 1/20 of the LC_50_) of PESE and PIC (Figure 1). The positive control induced a time-dependent decrease in survival, below 10%. In contrast, the negative control and the solvent control (0.1% DMSO) maintained high survival, mostly above 95%, with a slight decrease to 90% at the final time points. The glucose (2%) treatment caused moderate mortality, with survival decreasing to 75–85% over the course of the experiment. Exposure to the PESE at 4.0 and 2.0 μg/mL, survival consistently above 90%, with no indication of time-dependent decline. The higher concentration of the PIC (0.6 μg/mL; 2.5 μM) caused a modest reduction in survival to about 80%, whereas the lower dose (0.3 μg/mL; 1.3 μM) maintained viability above 90%.

**Figure 1:**
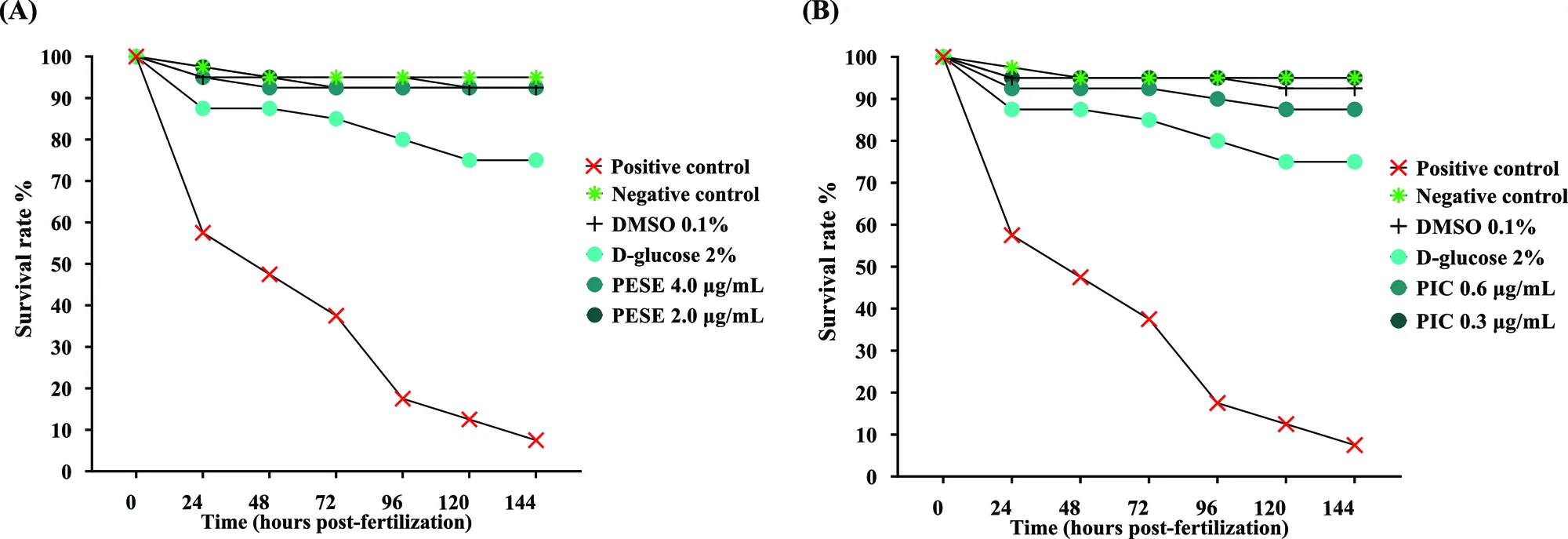
Survival of *Danio rerio* embryos exposed to 1/10 and 1/20 of the LC_50_ values of *P. edulis* seed ethanolic extract (PESE) and piceatannol (PIC) over 144 hpf. Control groups included negative control, solvent control (0.1% DMSO), *D*-glucose control (2%), and positive control (3,4-dichloroaniline). Tested concentrations were: (A) PESE at 4.0 μg/mL (1/10 LC_50_) and 2.0 μg/mL (1/20 LC_50_), and (B) PIC at 0.6 μg/mL (2.5 μM; 1/10 LC_50_) and 0.3 μg/mL (1.3 μM; 1/20 LC_50_). Survival was recorded at 0, 24, 48, 72, 96, and 144 hpf. Data are presented as mean ± SEM (*n* = 3).

Developmental abnormalities were assessed across multiple end points (Supporting Figure 5). PESE exposure induced minimal effects, with pericardial edema observed only at 2.0 μg/mL. In contrast, PIC elicited a broader spectrum of malformations, including pericardial edema and nonabsorption of the yolk sac, at both 0.3 μg/mL (1.3 μM) and 0.6 μg/mL (2.5 μM). The positive control caused a marked increase in frequency and severity of abnormalities, consistent with its known embryotoxicity, thus validating assay sensitivity. Mild alterations in the *D*-glucose 2% group reflected hyperglycemia-induced embryonic stress.

### 3.6 Glucose Modulation in Hyperglycemic Zebrafish Embryos

Total free glucose was quantified in zebrafish embryos under pulsatile (Figure 2) and continuous (Figure 3) hyperglycemia protocols. The pulsatile model, designed to mimic glycemic variability observed in gestational diabetes, induced marked glucose oscillations, with peaks at 24 and 72 hpf following d-glucose 2% exposure and reductions at 48 and 96 hpf during normoglycemic intervals. Control groups (embryo medium and 0.1% DMSO) maintained stable glucose levels throughout.

**Figure 2:**
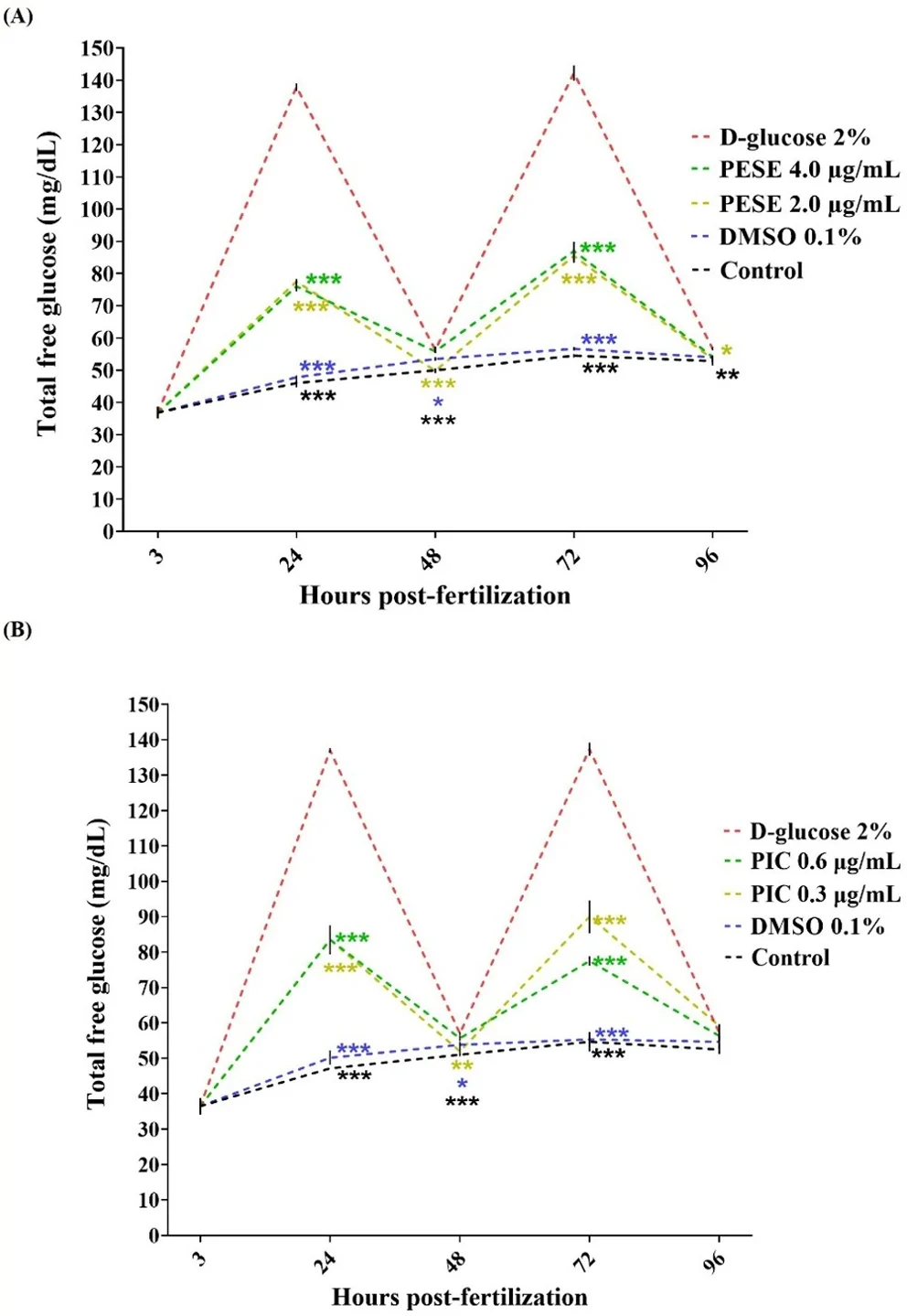
Antidiabetic effect of *P. edulis* seed ethanolic extract (PESE) and piceatannol (PIC) in a zebrafish model of pulsatile hyperglycemia. Glucose levels were measured at 3, 24, 48, 72, and 96 hpf. (A) PESE (2.0 and 4.0 μg/mL). (B) PIC (0.3 and 0.6 μg/mL; 1.3 and 2.5 μM). Data are presented as mean ± SEM. Statistical analysis was performed using one-way ANOVA followed by Dunnett’s post hoc test, comparing each treatment group with the hyperglycemic control (d-glucose 2%). Significance is indicated as *p < 0.05, **p < 0.01, ***p < 0.001. Symbols were color-coded according to treatment group.

**Figure 3:**
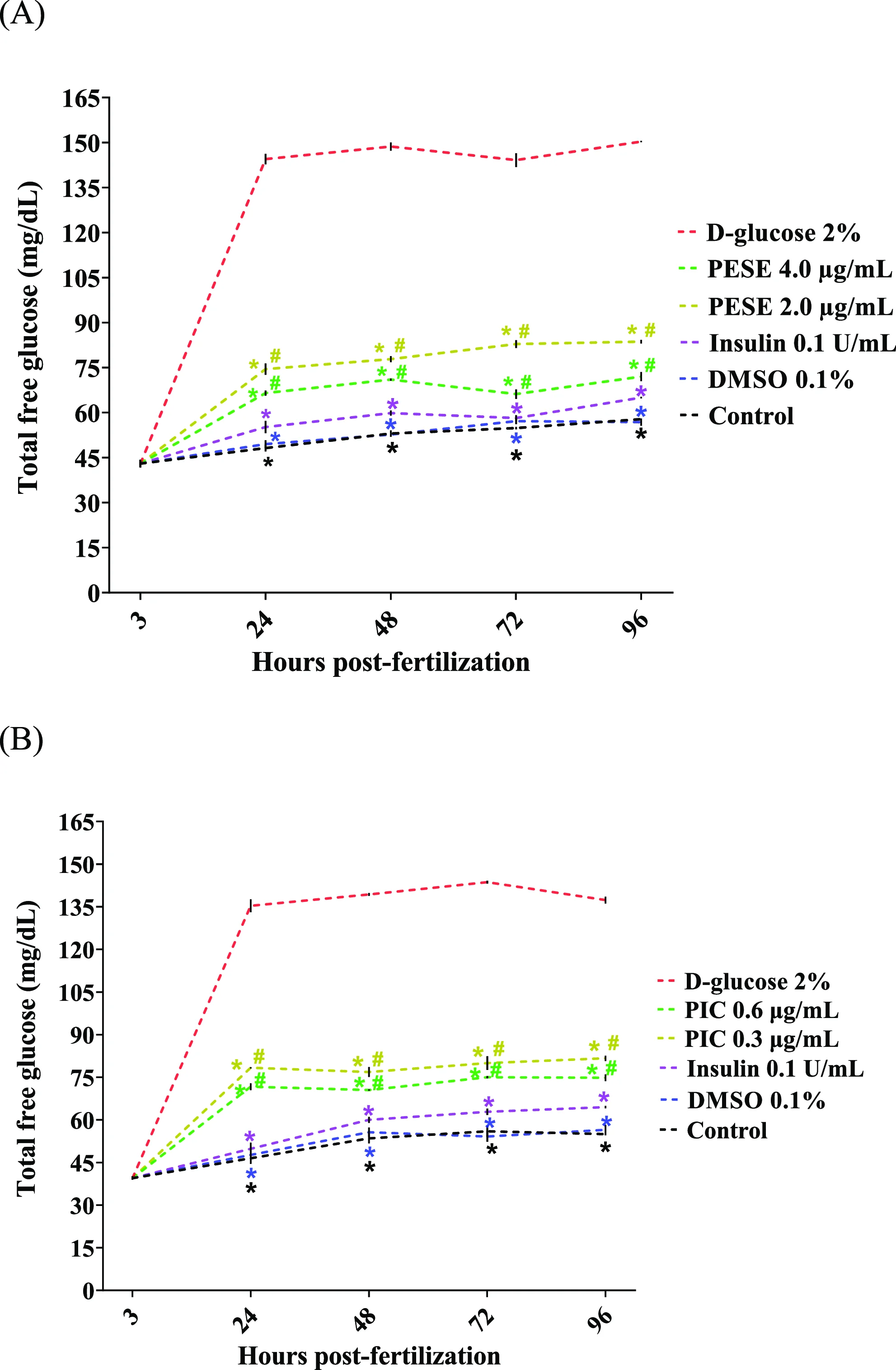
Effect of *P. edulis* seed ethanolic extract (PESE), piceatannol (PIC), and insulin on glucose levels in zebrafish embryos under continuous hyperglycemia. Embryos were exposed to d-glucose 2% and treated with (A) PESE (2.0 and 4.0 μg/mL) or (B) PIC (0.3 and 0.6 μg/mL; 1.3 and 2.5 μM), both compared with insulin (0.1 U/mL). Glucose was quantified at 3, 24, 48, 72, and 96 h. Data are expressed as mean ± SEM (*n* = 3). Statistical analysis was performed using two-way ANOVA, followed by Dunnett’s test vs *D*-glucose 2% and Tukey’s test vs. the insulin-treated group. Significance is indicated as *p ≤ 0.05 vs. *D*-glucose 2% and #*p* ≤ 0.05 vs. insulin (0.1 U/mL). Symbols were color-coded according to the treatment group.

All treatments (d-glucose 2% + PESE or PIC) significantly reduced glucose levels compared to the hyperglycemic group (*p* < 0.001). PIC showed a dose-dependent effect at 72 hpf, with the highest concentration (0.6 μg/mL; 2.5 μM) producing the greatest reduction. During normoglycemic intervals (48 and 96 hpf), the lowest concentrations of PESE (*p* < 0.001) and PIC (*p* < 0.01) restored glucose levels to values comparable to controls.

Under continuous hyperglycemia, *D*-glucose induced sustained glucose elevation from 24 hpf onward (*p* < 0.001). Insulin (0.1 U/mL) reduced glucose at all time points (*p* ≤ 0.05), confirming model sensitivity. PESE attenuated hyperglycemia at 4.0 μg/mL from 24 hpf, with a milder effect at 2.0 μg/mL, while PIC reduced glucose at both concentrations, with greater efficacy at 0.6 μg/mL (*p* ≤ 0.05). Although all treatments significantly lowered glucose compared to the hyperglycemic group (*p* ≤ 0.05), insulin remained more effective (*p* ≤ 0.05). Two-way ANOVA indicated a significant treatment–time interaction (*p* < 0.01).

### 3.7 Oxidative Stress and Antioxidant Gene Expression in Zebrafish Embryos

Continuous exposure of zebrafish larvae to *D*-glucose 2% resulted in a marked elevation in ROS production, reaching nearly a 3-fold increase compared to the control (*p* ≤ 0.05). Both control groups (embryo medium and 0.1% DMSO) maintained basal ROS levels, with no significant differences between them. Treatment with PESE (4.0 and 2.0 μg/mL) and PIC (0.6 and 0.3 μg/mL) significantly attenuated glucose-induced ROS generation (*p* ≤ 0.05), although elevated compared to the controls (Figure 4).

**Figure 4:**
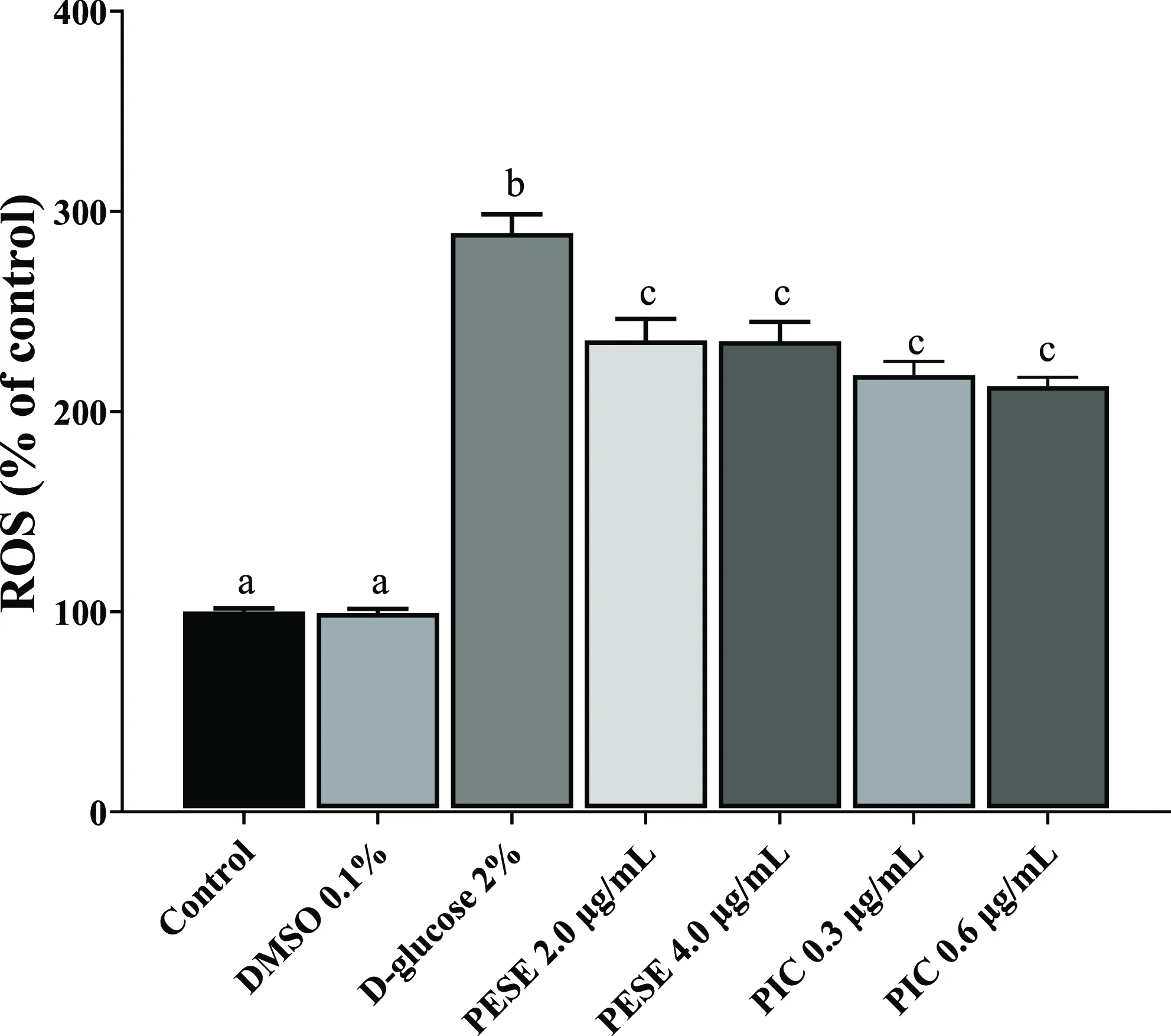
Effect of *P. edulis* seed ethanolic extract (PESE) and piceatannol (PIC) on reactive oxygen species (ROS) production in zebrafish larvae exposed to continuous hyperglycemia. ROS levels were measured after 96 hpf to d-glucose 2%. Control group: embryo medium. Data is presented as mean ± SEM. Statistical analysis was performed using one-way ANOVA followed by Tukey’s post hoc test. Different letters indicate significant differences (*p* ≤ 0.05).

Continuous exposure to *D*-glucose 2% significantly altered the expression of antioxidant genes in zebrafish larvae, with increased levels of *sod2, gpx1a*, and *nfe2l2a* (*p* ≤ 0.05) and decreased expression of *sod1* and *cat* (*p* ≤ 0.05), while *gsr* remained unchanged (*p* > 0.05) (Figure 5). PESE markedly enhanced *nfe2l2a* expression, particularly at 2.0 μg/mL (∼5-fold), accompanied by significant upregulation of *sod1, sod2*, and *cat* (*p* ≤ 0.05). PIC also induced strong *nfe2l2a* activation, most evident at 0.3 μg/mL (∼4-fold), together with significant increases in *gpx1a* and *cat* (*p* ≤ 0.05). At higher concentrations, PIC maintained positive but less pronounced effects. Overall, PESE at 2.0 μg/mL and PIC at 0.3 μg/mL elicited the most robust activation of the Nrf2 pathway, accompanied by coordinated modulation of downstream antioxidant genes.

**Figure 5:**
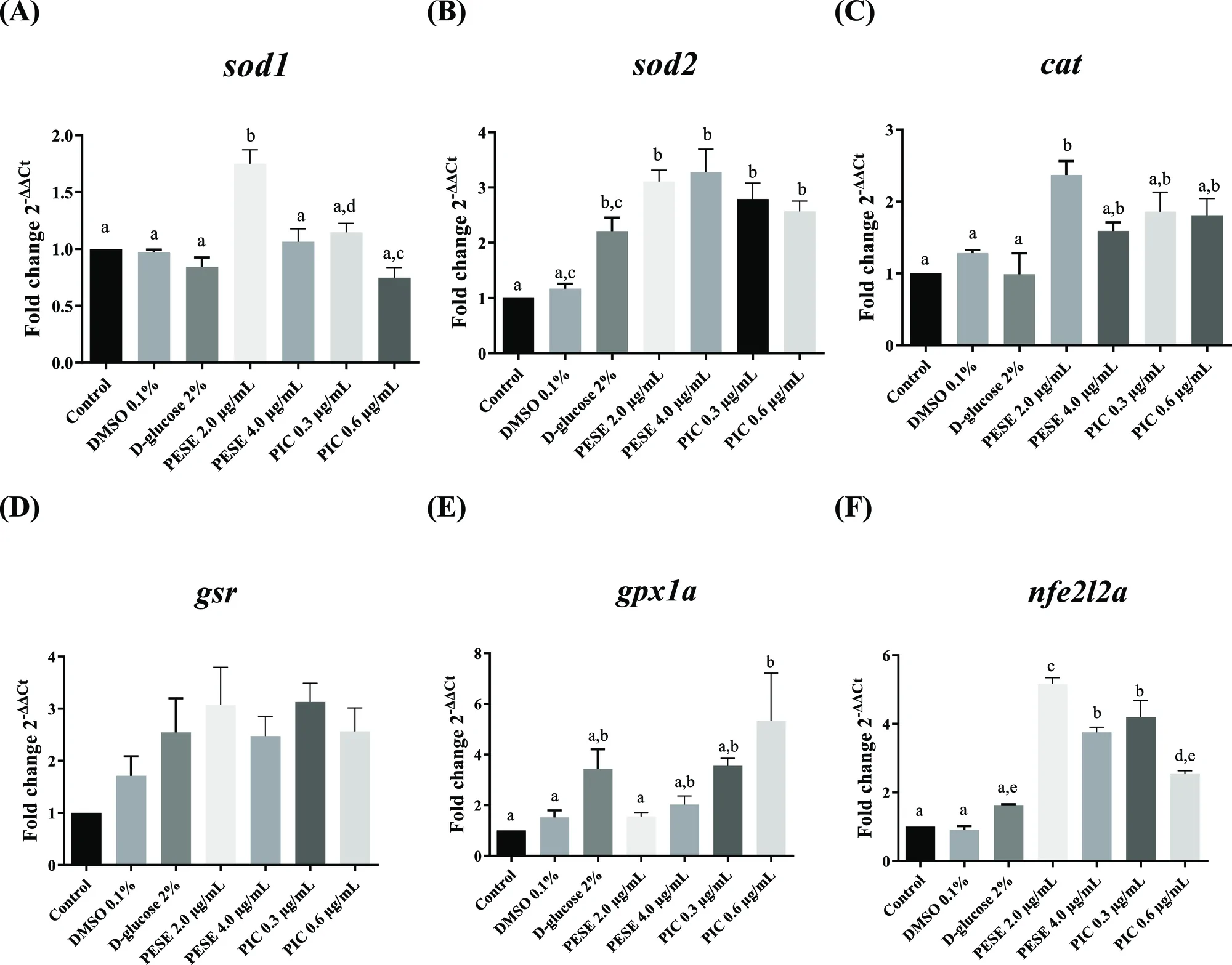
Relative expression of oxidative stress-related genes in zebrafish embryos exposed for 96 hpf to DMSO (0.1%), *D*-glucose (2%), PESE (4.0 and 2.0 μg/mL), and PIC (0.3 and 0.6 μg/mL; 1.3 and 2.5 μM). Genes analyzed were (A) superoxide dismutase 1 (*sod1*), (B) superoxide dismutase 2 (*sod2*), (C) catalase (*cat*), (D) glutathione-disulfide reductase (*gsr*), (E) glutathione peroxidase 1a (*gpx1a*), and (F) nuclear factor erythroid 2-related factor 2 (*nfe2l2a*). Data are expressed as mean ± SEM. Statistical analysis was performed using one-way ANOVA followed by Tukey’s post hoc test. Different letters indicate significant differences (*p* ≤ 0.05).

## 4 Discussion

The valorization of agro-industrial residues as sources of bioactive compounds represents a sustainable strategy with direct implications for human health. In this study, we identified PIC as a constituent of PESE and demonstrated that both PESE and PIC exert significant antioxidant and antidiabetic effects. These findings, observed in complementary in vitro and in vivo models, highlight their potential therapeutic relevance for T2DM and GDM, integrating metabolic control with sustainable innovation.

High-resolution mass spectrometry analysis of PESE revealed the presence of phenolic compounds of the stilbene and flavonoid classes, including PIC, isookanin-7-*O-*gludoside, scirpusin B, and naringenin, with annotations based on characteristic fragmentation patterns. Among these metabolites, stilbenes constitute a recurrent group in *P. edulis* seeds, notably PIC, widely recognized for its biological properties, and scirpusin B, a PIC-derived dimer. Previous studies corroborate our findings by identifying PIC as a principal constituent of this matrix, whose levels are strongly influenced by the extraction method, particularly in acetone- or ethanol-based protocols. Notably, PIC occurs in higher concentrations and exhibits enhanced bioactivity, attributed to the presence of an additional *ortho*-hydroxyl group (catechol motif) in its structure, which enhances its antioxidant and anti-inflammatory properties.^11,12,15,42–46^

PESE presented a high total phenolic content (238.9 ± 1.9 mg GAE/g dry extract), confirming passion fruit seeds as a phenolic-rich matrix. This value is consistent with previous reports describing high TPC in *P. edulis* seeds, ranging from 250 to 332 mg GAE/g DW, depending on extraction conditions.^47–49^

The literature reports a direct association between TPC and antioxidant activity in plant matrices,^15,47–49^ which may explain the significant antioxidant capacity observed for PESE, particularly against HOCl and O_2_^•–^. In parallel, PIC showed greater activity in DPPH^•^ and HOCl assays. Similar antioxidant profiles for *P. edulis* seed extracts and isolated PIC have been reported, with IC_50_ values comparable to those observed in the present study.^15^

PTP1B is a negative regulator of insulin and leptin signaling pathways and represents a relevant therapeutic target for T2DM and obesity. It dephosphorylates the insulin receptor and IRS proteins, attenuating insulin signaling, and also dephosphorylates JAK2, reducing LEPR–JAK2–STAT signaling and indirectly promoting appetite via increased NPY expression.^18,50–52^

In the present study, only PESE exhibited significant inhibitory activity against PTP1B (IC_50_ = 29.8 ± 2.7 μg/mL), achieving an effective concentration nearly twice that of the positive control ursolic acid. In contrast, isolated PIC showed no activity up to the highest concentration tested (2.5 μg/mL). This clear difference strongly suggests that other metabolites present in the extract, such as scirpusin B, naringenin, or yet unidentified compounds, are primarily responsible for the PTP1B inhibitory effect.^53,54^

This result highlights a key advantage of the whole extract over the isolated compound: the multitarget potential offered by the complex phytochemical matrix of PESE. While PIC demonstrated superior potency in several assays (e.g., α-glucosidase inhibition, oxidative burst suppression, and glucokinase activation), the crude extract exhibited broader activity, including relevant inhibition of PTP1B. Such complementary actions may provide more comprehensive therapeutic benefits for managing the multifaceted pathophysiology of diabetes mellitus. For PIC, the literature remains controversial, with reports describing either no measurable inhibition or potent activity, likely due to methodological differences.^53,54^ These inconsistencies highlight the need for standardized assays to clarify its inhibitory potential against PTP1B.

GK catalyzes the phosphorylation of glucose to glucose-6-phosphate and plays a central role in glucose sensing and glycemic homeostasis, making its activation a therapeutic strategy for T2DM. In this study, both PESE and PIC activated GK, with PIC showing higher potency than PESE but markedly lower activity than the positive control dorzagliatin. Nevertheless, PIC exhibited a measurable modulatory effect on GK activity, and to our knowledge, this is the first study directly evaluating the effects of PIC and PESE on GK.^55,56^

Regarding nonhuman antidiabetic enzymes, both PESE and PIC inhibited α-amylase and α-glucosidase. PIC showed a favorable selectivity profile, with stronger inhibition of α-glucosidase than α-amylase, a pattern considered advantageous for controlling postprandial glycemia. Previous studies with *Passiflora* species reported moderate inhibition of both enzymes, while *P. edulis* seed extracts showed greater α-amylase inhibition. Isolated PIC stood out for α-glucosidase inhibition, indicating enzyme-specific modulation by individual compounds.^15,57^

Overall, the results confirm the importance of *Passiflora* agroindustrial residues as a source of bioactive agents for hyperglycemia management. Variability in inhibitory profiles across species and matrices may be attributed to differences in polyphenol composition, which influence the selectivity and intensity of enzyme inhibition.^57,58^

In the present study, the neutrophil oxidative burst was evaluated using luminol and APF oxidation assays. In the luminol assay, PIC showed the highest inhibitory effect, comparable to VAS2870 (positive control), followed by *P. edulis* seed extract, which also significantly inhibited oxidative burst, although to a lesser extent.

Luminol detects a broad range of reactive oxygen and nitrogen species and is commonly used to assess neutrophil oxidative activity.^22,30^ To date, no studies have specifically evaluated the effects of *P. edulis* seed extract on human neutrophil oxidative burst, highlighting the novelty of the present findings. Previous reports have shown inhibitory effects of PIC on luminol oxidation at micromolar concentrations,^59,60^ while other plant extracts show variable activity.^61^ In the APF assay, which selectively detects hypochlorous acid formation during the oxidative burst, both *P. edulis* seed extract and PIC effectively reduced HOCl levels.

Taken together, these findings highlight the relevance of investigating agents that modulate neutrophil oxidative burst, as dysregulated reactive species production is directly implicated in metabolic dysfunction. In T2DM, characterized by chronic oxidative stress, redox-modulating compounds may contribute to improved glycemic control and attenuation of diabetes-related complications.^14^

The toxicological evaluation in *Danio rerio* embryos revealed distinct profiles for PESE and PIC. PESE showed lower toxicity and a wider margin of tolerance, whereas PIC induced progressive mortality, with total lethality at high concentrations (PESE: 100 μg/mL; PIC: 19 μg/mL/77.8 μM). At elevated concentrations, both samples caused atypical pigmentation and hatching inhibition, despite preserved cardiac activity and stable experimental conditions.

These effects may be associated with oxidative conversion of PIC into colored quinones and adsorption of phenolic compounds to the chorion, impairing permeability and hatching enzyme activity. Similar interactions have been shown to disrupt protease secretion, chorion digestion, and oxygen diffusion during embryogenesis.^62–66^

Given that PIC is the major secondary metabolite in PESE, the concentration-dependent effects observed are likely related to its intrinsic properties. Although recognized for antioxidant activity, polyphenols may exhibit pro-oxidant behavior at high concentrations, which may explain the embryotoxicity observed. Accordingly, PESE presented a higher safety index than PIC, with concentrations below 9.5 μg/mL (38.9 μM) considered safe for biological applications.

The LC_50_-to-active-concentration ratios were approximately 10.3–20.7 for PIC and 9.8–19.7 for PESE. These values indicate a reasonable separation between concentrations that induce acute lethality and those producing observable biological activity in the zebrafish embryo model. However, these ratios should not be interpreted as formal therapeutic indices or as direct evidence of clinical safety. A 10–20-fold margin in an embryonic screening model is promising for a natural product but remains insufficient for nutraceutical or therapeutic translation. Notably, PIC induced sublethal developmental alterations (e.g., pericardial edema and yolk sac nonabsorption) at concentrations close to the effective range. Therefore, further studies including repeated-dose toxicity, developmental and reproductive toxicology, genotoxicity, pharmacokinetics, and efficacy/safety evaluation in mammalian models will be essential before establishing a safe therapeutic window.

Phenolic compounds such as PIC are synthesized by plants as adaptive defense responses and are valued for their nutraceutical properties in human health. Thus, the toxicity observed in zebrafish embryos does not negate therapeutic potential but emphasizes the importance of dose definition and biological context.^67^

In preclinical research, toxicity assessment is a recommended initial step, commonly performed in zebrafish embryos using the LC_50_ as a reference, followed by testing concentrations 10–100-fold lower.^32,33^ Under this framework, PESE showed a more favorable toxicological profile than PIC, whereas PIC requires careful risk–benefit evaluation.

Morphological analysis reinforced these differences. PESE at 4.0 and 2.0 μg/mL caused minimal developmental alterations, while PIC at 0.3 and 0.6 μg/mL (1.3 and 2.5 μM) induced changes in multiple structures, indicating greater embryotoxicity. Such alterations are consistent with reports that phenolic compounds, despite their antioxidant properties, may exert pro-oxidant effects at higher concentrations, leading to cellular damage and impaired embryonic development.^64–66^

Analysis of glycemic modulation in zebrafish embryos showed that both PESE and PIC significantly attenuated glucose elevations under alternating and continuous hyperglycemic regimens. In the continuous exposure model, their effects were comparable or close to those of insulin, reinforcing their potential as adjunctive agents for hyperglycemia management. The differential responses between regimens indicate that exposure patterns influence efficacy, with continuous exposure favoring glycemic stability, while pulsatile administration may be relevant for intermittent or controlled-release strategies.^23,68,69^

Zebrafish is a well-established model for hyperglycemia studies due to its translational relevance and responsiveness to antidiabetic compounds. Sustained exposure to d-glucose 2% has been shown to induce marked hyperglycemia, while treatment with bioactive compounds, including flavonoids, reduces oxidative stress and improves antioxidant defenses.^68,69^

Flavonoids and stilbenes present in PESE exhibit antioxidant, anti-inflammatory, and antiglycation properties, contributing to glycemic control through radical scavenging, modulation of inflammatory pathways, and protection of pancreatic β-cells.^70–72^ Similar hypoglycemic effects have been reported for phenolic-rich plant extracts in zebrafish hyperglycemia models, supporting the role of phenolic compounds in reducing glucose levels.^73,74^

Consistent with the link between hyperglycemia and oxidative stress, embryos exposed to glucose showed increased ROS levels, whereas PESE and PIC significantly reduced ROS release at all tested concentrations. This dual hypoglycemic and antioxidant effect highlights their potential relevance for managing diabetes mellitus, particularly given the contribution of oxidative stress to diabetic complications.

Excessive ROS production beyond cellular antioxidant capacity leads to oxidative damage and may trigger programmed cell death. Metabolically active tissues generate high ROS levels and are therefore particularly vulnerable in conditions such as DM, which is closely associated with oxidative stress. This vulnerability is partly attributed to the intrinsically low antioxidant enzyme activity in pancreatic β-cells, supporting the use of Danio rerio as a relevant model for investigating metabolic dysfunctions and therapeutic interventions targeting oxidative stress.^75^

To further explore antioxidant modulation, the expression of redox-related genes (*sod1, sod2, cat, gsr, gpx1a, and nfe2l2a*) was evaluated. PESE significantly upregulated *sod1, sod2, cat*, and *nfe2l2a*, indicating coordinated activation of superoxide dismutation and hydrogen peroxide detoxification pathways under Nrf2 regulation. In contrast, PIC showed a more restricted response, increasing *nfe2l2a, gpx1a*, and *cat*, suggesting preferential modulation of glutathione- and peroxidase-dependent mechanisms. These differences likely reflect variations in chemical composition and the diversity of secondary metabolites present in each sample.^35^

Notably, both treatments increased *nfe2l2a* expression, which encodes the Nrf2 transcription factor, a key regulator of antioxidant and detoxification responses. This transcriptional response is consistent with modulation of the Nrf2/ARE pathway, which regulates genes encoding classical antioxidant enzymes, including SOD, CAT, and GPx, as well as phase II detoxification enzymes. This response is consistent with previous evidence showing that phenolic compounds can modulate Nrf2 signaling, with complex extracts such as PESE potentially promoting broader cytoprotective responses than isolated compounds.^35,76,77^

### 4.1 Translational Relevance and Therapeutic Application

The comprehensive antidiabetic and antioxidant profile demonstrated by PESE and PIC in human and nonhuman enzymatic assays, human neutrophils, and the zebrafish hyperglycemia model highlights their potential as multifunctional agents for the management of T2DM and GDM. These preparations act on multiple pathophysiological targets simultaneously, including inhibition of α-amylase and α-glucosidase, glucokinase activation, PTP1B inhibition (by PESE), suppression of neutrophil oxidative burst, reduction of hyperglycemia-induced ROS production, and Nrf2-mediated antioxidant gene expression, addressing both hyperglycemia and oxidative stress, which are not fully controlled by current standard therapies.

As passion fruit seeds are an abundant and low-cost agro-industrial byproduct, PESE emerges as a sustainable candidate for its development as a dietary supplement or functional food ingredient, especially for preventive strategies or as an adjunct therapy to conventional antidiabetic drugs (e.g., metformin or acarbose). PIC, the major bioactive, showed superior potency in most assays and could be explored as a purified compound for more targeted formulations.

Future translational efforts should prioritize: (i) pharmacokinetic and bioavailability studies in rodents and humans; (ii) efficacy and safety evaluation in rodent models of T2DM and GDM; (iii) chronic and reproductive toxicity assessments; (iv) investigation of possible synergistic effects with existing antidiabetic medications; and (v) formulation strategies (such as nanoencapsulation) to improve the stability and absorption of PIC.

If these steps confirm the present findings, PESE and PIC-enriched products could represent a promising, natural, and cost-effective adjunctive approach for diabetes management while promoting the valorization of agro-industrial waste.

### 4.2 Limitations

Despite the promising results obtained with PESE and PIC, some methodological limitations must be considered. The lack of validation in superior models limits the direct extrapolation of the observed effects to mammalian systems, including humans. It requires caution and further investigation in more complex in vivo models. In addition, the study was limited to acute toxicity, no chronic approaches or long-term behavioral, genotoxic, or reproductive assessments were performed, which are crucial for a complete characterization of therapeutic safety.

Another limitation is the lack of quantitative determination of PIC and other major phenolic compounds in PESE. However, despite identifying PIC as a major constituent, we are unable to determine its precise contribution to the observed biological effects of the extract. Therefore, the superior or complementary activity of PESE compared to isolated PIC may result from synergistic interactions with other metabolites such as scirpusin B and naringenin (entourage effect). Future studies should include validated quantitative LC-MS/MS methods to clarify the relative contribution of each compound.

Furthermore, the present enzyme assays demonstrate functional modulation but do not establish direct ligand–target binding sites or molecular interaction mechanisms. Molecular docking and molecular dynamics simulations were not performed because the present study was designed to provide a direct experimental comparison between PESE and PIC across biochemical, cellular, and whole-organism models, rather than to propose specific binding poses or direct molecular interactions. In addition, PESE is a chemically complex mixture that has not yet been quantitatively standardized, which prevents the evidence-based selection of the constituent or combination of constituents responsible for each enzymatic effect. This is particularly relevant for PTP1B, which was inhibited by PESE, whereas isolated PIC showed no measurable inhibitory activity within the tested concentration range. Future investigations should therefore combine quantitative chemical characterization, bioactivity-guided fractionation, enzyme-kinetic studies, purified-compound testing, molecular docking, and molecular dynamics simulations to identify the compounds responsible for each target-specific response and avoid unsupported mechanistic attribution.

In this context, the *Danio rerio* embryonic model has proven to be an efficient, sustainable, and cost-effective tool for screening toxic, antioxidant, and antidiabetic effects, providing relevant data for the development of safe nutraceutical and herbal formulations. Of note are PESE and PIC, which demonstrated not only a favorable safety profile but also a significant ability to reduce blood glucose in models of hyperglycemia, associated with the induction of antioxidant enzymes via activation of the Nrf2/ARE pathway. These findings position these compounds as strategic candidates for controlling oxidative stress and preventing metabolic disorders such as DM.

From a scientific perspective, this research enhances understanding of the biological activity in lesser-used plant components and proposes sustainable biotechnological approaches to repurpose agro-industrial waste. It also supports a scientific agenda rooted in circular economic principles by converting environmental burdens into beneficial resources with public health benefits. To build on these findings, further investigations are recommended, including metabolomic profiling, mammalian preclinical testing, and advanced delivery techniques such as encapsulation or nanocarriers, to improve compound stability and absorption. These steps will help translate the research into practical applications across the pharmaceutical, food, and environmental sectors, underscoring its strategic and innovative significance.

## 5 Conclusion

This study demonstrated that *Passiflora edulis* seed ethanolic extract (PESE) and isolated PIC exhibit distinct but complementary antioxidant and antidiabetic profiles across chemical, enzymatic, cellular, and embryonic zebrafish models. PIC showed greater potency in several antioxidant assays, inhibition of α-amylase and α-glucosidase, glucokinase modulation, and suppression of the human neutrophil oxidative burst. In contrast, PESE displayed broader multitarget activity, including PTP1B inhibition, suggesting that other constituents of the extract may contribute to its biological effects.

Both PESE and piceatannol attenuated hyperglycemia-associated oxidant production and modulated antioxidant gene expression in zebrafish embryos. However, the toxicological findings revealed a more favorable embryonic safety profile for PESE than for isolated PIC, emphasizing the importance of dose selection and comprehensive safety assessment.

Overall, the direct comparison between the whole extract and the isolated compound provides new evidence that the biological activity of PESE cannot be attributed exclusively to piceatannol. These findings support the potential of passion fruit seeds as a sustainable source of multifunctional bioactive compounds, while reinforcing the need for chemical standardization, quantitative characterization, pharmacokinetic studies, and further evaluation of efficacy and safety in mammalian models before translational or nutraceutical applications can be considered.

## Supplementary Material



## Notes

ESI­(−) fragmentation profile for the compound piceatannol (*m*/*z* 243.07) (Figure S1); ESI(−) fragmentation profile for the compound scirpusin B (*m*/*z* 485.12) (Figure S2); ESI(−) fragmentation profile for the compound naringenin (*m*/*z* 271.06) (Figure S3); L*C*_min_, LC50, L*C*_max_ values and sublethal concentrations derived from the compounds tested in *Danio rerio* embryos after 96 hpf (Table S1); primer sequences for gene expression analysis and associated parameters (Table S2); physicochemical data of the embryo medium after exposure to different concentrations of treatments (Table S3); and total phenol content (TPC) and antioxidant capacity of *P. edulis* seed ethanolic extract and PIC (Table S4) (PDF)

## Abbreviations

ABAH: 4-aminobenzylhydrazine
α-Amylase: porcine pancreatic α-amylase
α-Glucosidase: *Saccharomyces cerevisiae* α-glucosidase
ANOVA: analysis of variance
APF: 2-[6-(4′-amino)­phenoxy-3H-xanthen-3-on-9-yl]­benzoic acid
ATP: adenosine triphosphate
BSA: bovine serum albumin
β-NADH: β-nicotinamide adenine dinucleotide
CNPG3: 2-chloro-4-nitrophenyl-α- *D* -maltotrioside
DM: diabetes mellitus
DHR-123: dihydrorhodamine-123
DMSO: dimethyl sulfoxide
DPPH^•^: 2,2-diphenyl-1-picrylhydrazyl radical
DTT: dithiothreitol
FC: folin-ciocalteu
FET: fish embryo acute toxicity test
GAE: gallic acid equivalents
GDM: gestational diabetes
GK: glucokinase
H_2_DCFDA: 2′,7′-dichlorodihydrofluorescein diacetate
hpf: hours postfertilization
IC_50_: half-maximum inhibitory concentration
LC50: lethal concentration 50%
LC–MS: liquid chromatography–mass spectrometry
MIQE: minimum information for publication of quantitative real-time PCR experiments
NBT: p-nitroblue tetrazolium chloride
NOAEL: no observed adverse effect level
OECD: organisation for economic co-operation and development
PBS: Dulbecco’s phosphate-buffered saline
PESE: *Passiflora edulis* seed ethanolic extract
PIC: piceatannol
PMS: *N*-methylphenazonium methyl sulfate
PMA: phorbol-12-myristate-13-acetate
PNPG: 4-nitrophenyl-β-d-glucuronide trisodium
PTP1B: protein tyrosine phosphatase 1B
pNPP: p-nitrophenyl phosphate
qPCR: quantitative real-time polymerase chain reaction
RONS: reactive oxygen and nitrogen species
SEM: standard error of the mean
T2DM: type 2 diabetes mellitus
TPC: total phenol content.
