# Supplementary material for: Evaluation of Passiflora edulis Seed Extract and Piceatannol in Attenuating Hyperglycemia and Oxidative Stress Using Human and Nonhuman Antidiabetic Enzymes, Human Neutrophil Oxidative Burst, and Embryonic Zebrafish Hyperglycemia Model
Source: ACS Omega. 2026 Sep 14;11(38):56963–78. doi: 10.1021/acsomega.6c03845 (PMC13628997; doi:10.1021/acsomega.6c03845)
Supplement: Supplementary file 1 [file ao6c03845_si_001.pdf]

Supporting Information

Evaluation of *Passiflora edulis* Seed Extract and Piceatannol in Attenuating Hyperglycemia and Oxidative Stress Using Human and Nonhuman Antidiabetic Enzymes, Human Neutrophil Oxidative Burst, and Embryonic Zebrafish Hyperglycemia Model

Elaine L. S. S. Mendonça; Ana Ramalho; Boniek G. Vaz; Alane C. M. Oliveira;

Jadriane A. Xavier; Marisa Freitas; Eduarda Fernandes; Marília O. F. Goulart**

Contents:

Figure S1. ESI(-) fragmentation profile for the compound piceatannol (m/z 243.07).
Figure S2. ESI(-) fragmentation profile for the compound scirpusin B (m/z 485.12).
Figure S3. ESI(-) fragmentation profile for the compound naringenin (m/z 271.06).
Table S1. LCmin, LC50, LCmax values and sublethal concentrations derived from the compounds tested in Danio rerio embryos after 96 hpf.
Table S2. Primer sequences for gene expression analysis and associated parameters
Table S3. Physicochemical data of the embryo medium after exposure to different concentrations of treatments.
Table S4. Total phenol content (TPC) and antioxidant capacity of P. edulis seed ethanolic extract and PIC

Number of pages: 9

Number of tables: 4

Number of figures: 3

Figure S1. ESI(-) fragmentation profile for the compound piceatannol (*m/z* 243.07).

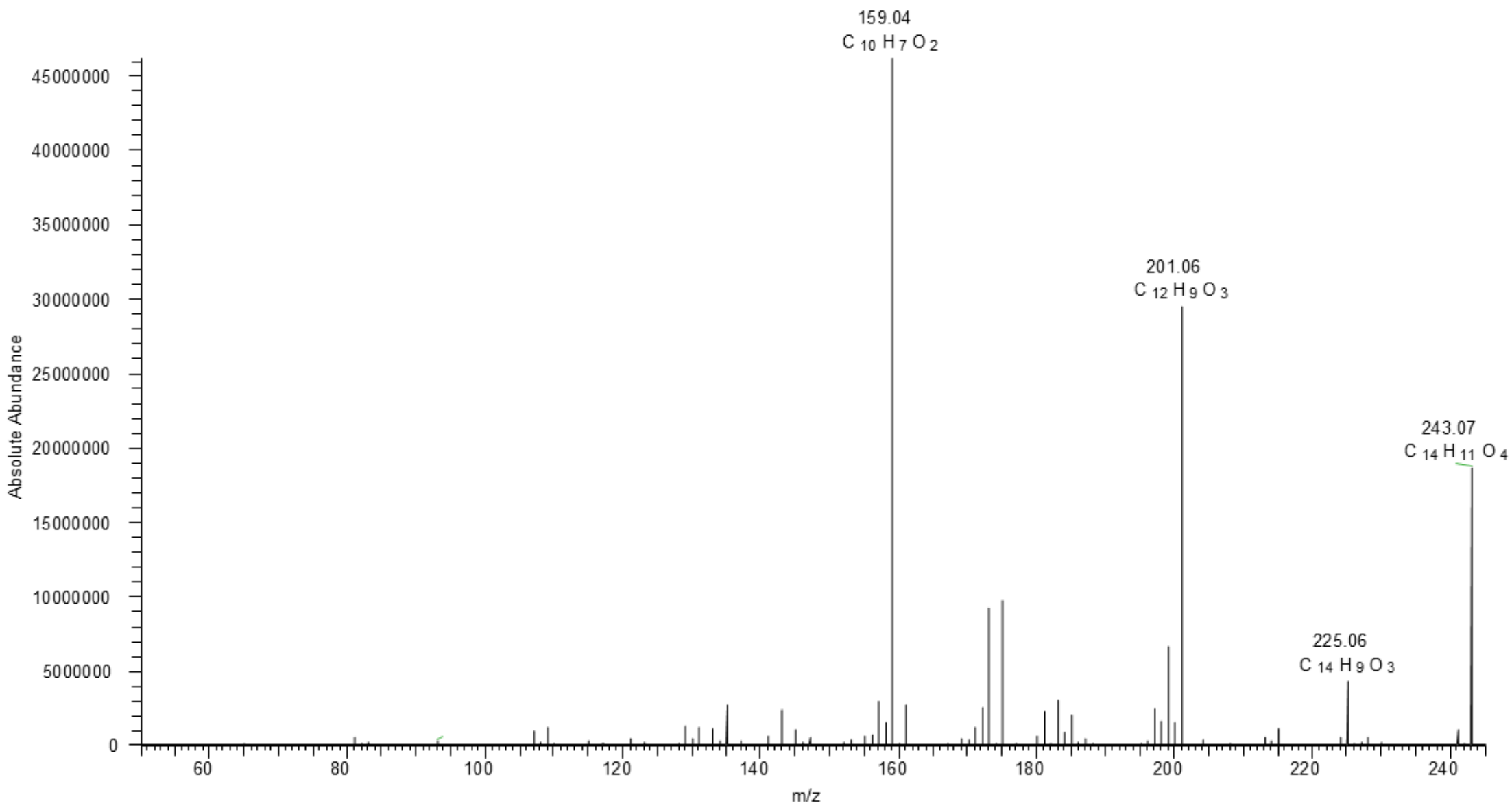

Figure S2. ESI(-) fragmentation profile for the compound scirpusin B (*m/z* 485.12).

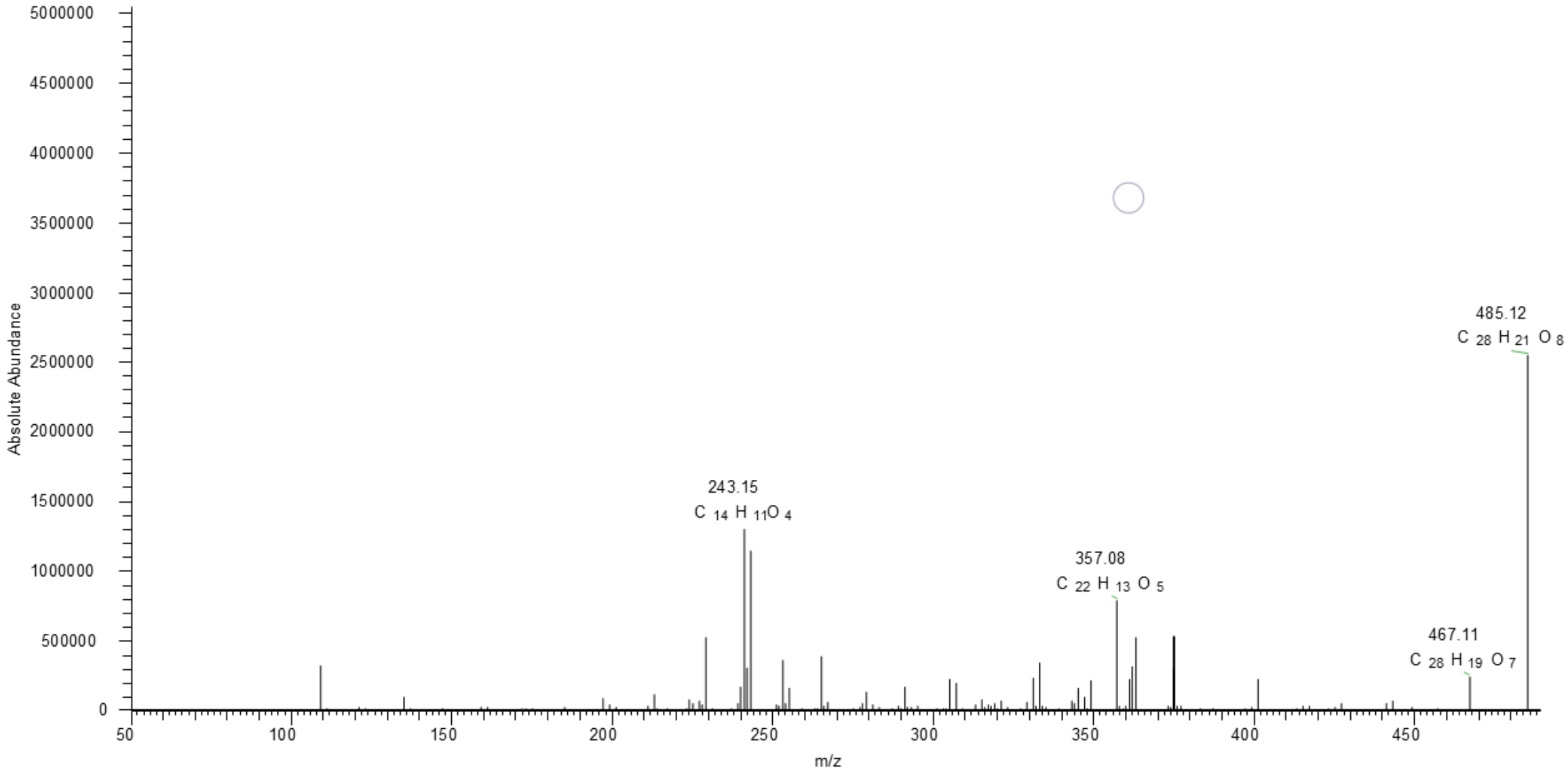

Figure S3. ESI(-) fragmentation profile for the compound naringenin (*m/z* 271.06).

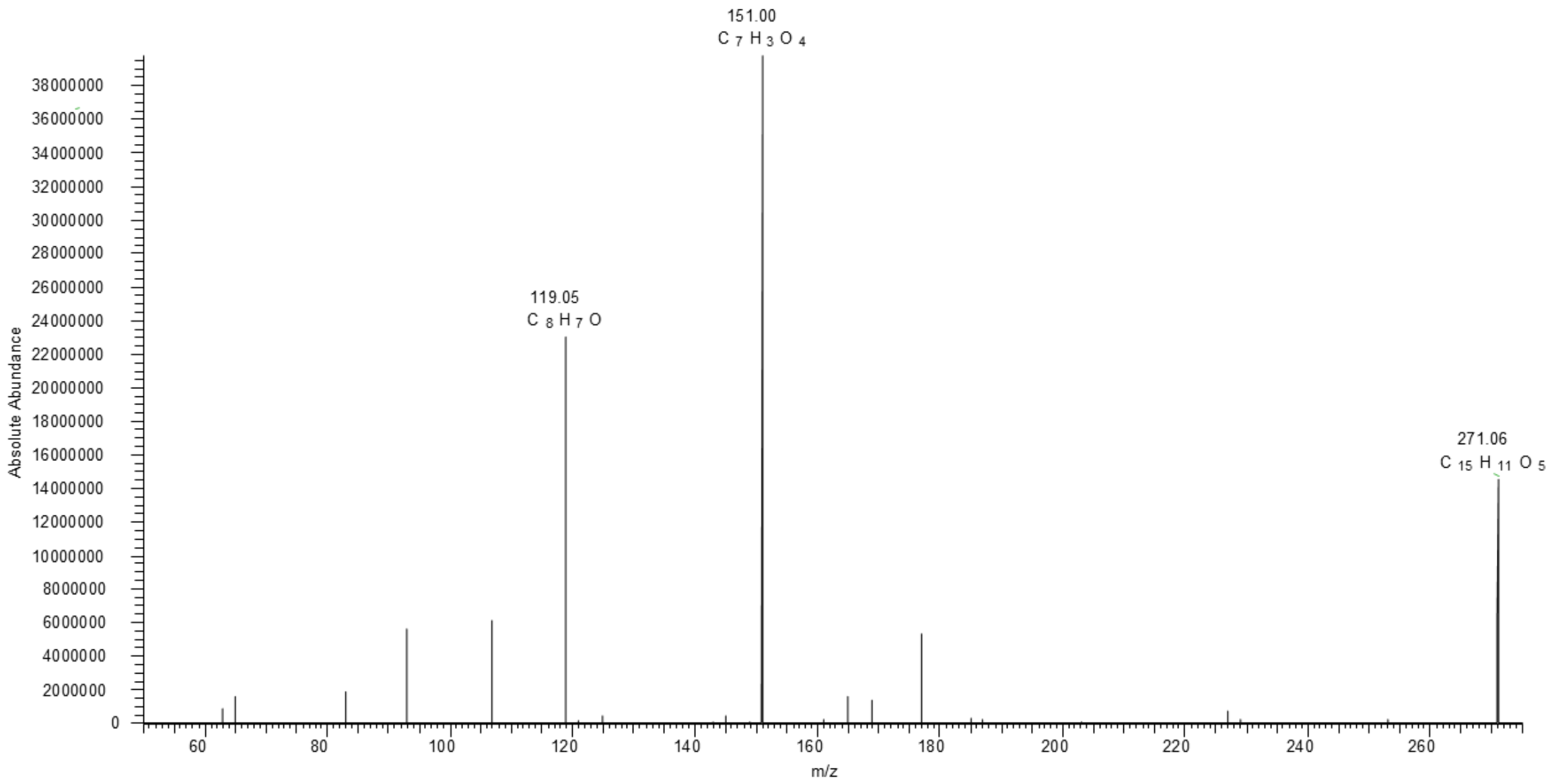

Table S1. LC_{min}, LC_{50}, LC_{max} values and sublethal concentrations derived from the compounds tested in *Danio rerio* embryos after 96 hpf.

**Treatment**	**LC_{min}**	**LC_{50}**	**LC_{max}**	**$\frac{1}{20}LC_{50}$**	**$\frac{1}{10}LC_{50}$**
**PESE**	6.2 µg/mL	39.3 µg/mL	100 µg/mL	2.0 µg/mL	4.0 µg/mL
**PIC**	1.2 µg/mL (4.9 µM)	6.2 µg/mL (25.4 µM)	19 µg/mL (77.8 µM)	0.3 µg/mL (1.3 µM)	0.6 µg/mL (2.5 µM)

PESE: *P. edulis* seed ethanolic extract; PIC: piceatannol; LCmin: minimum lethal concentration; LCmax: maximum lethal concentration; LC50: median lethal concentration.

Table S2. Primer sequences for gene expression analysis and associated parameters

Gene	**Database Accession Number**	**Primer Forward (5’–3’)**	**Primer Reverse (5’–3’)**	**Size (bp)**	**Melting Temperature (°C)**
sod1 [1]	NM_131294.1	GTAATGTGACCGCTGATGCC	GAATCACCATGGTCCTCCCA	105	60
sod2 [1]	NM_199976.1	TTGGAGGCCATAAAGCGTGA	CCTTTTCAAAGCCCAGCCAG	112	60
cat [1]	NM_130912.2	TCTCCTGATGTGGCCCGATA	GGTTTTGCACCATGCGTTTC	169	60
gpx1a [1]	NM_001007281.2	TAAAACCTGCGTGTTGCCCT	ATAGTTTCGCGGACAGGTCG	97	60
gsr [1]	NM_001020554.1	TCCAGAACACCTCTCGTCCA	ACTGCTTCATCAGGTGTCAGA	79	60
nfe2l2a[1]	NM_182889.1	TGGCCCTGAAGAATTTAACG	CCCGGTGAGAAGCTCTGTAG	138	60
actb [2]	NM_181601.5	CTGTTCCAGCCATCCTTCTT	TGTTGGCATACAGGTCCTTAC	109	60

[1-](Junior et al., 2021); [2-] (Li et al., 2019)

Table S3. Physicochemical data of the embryo medium after exposure to different concentrations of treatments.

Concentration (µg/mL)	Temperature (°C)	pH
**PIC**		
0.60	26.00 ± 0.10	7.20 ± 0.10
1.20	26.12 ± 0.15	6.95 ± 0.13
2.40	26.06 ± 0.18	6.93 ± 0.13
4.80	26.07 ± 0.11	6.82 ± 0.07
9.60	26.13 ± 0.22	6.79 ± 0.04
19.00	26.07 ± 0.14	6.94 ± 0.11
**PESE**		
6.20	26.12 ± 0.17	6.91 ± 0.17
12.50	26.18 ± 0.20	6.70 ± 0.13
25.00	26.19 ± 0.21	6.69 ± 0.07
50.00	26.00 ± 0.06	6.72 ± 0.07
100.00	26.12 ± 0.17	6.72 ± 0.21

Mean ± SD.

Table S4. Total phenol content (TPC) and antioxidant capacity of *P. edulis* seed ethanolic extract and PIC

Sample	TPC (mg GAE/g DE)	IC_{50} µg/mL (µM)		
		DPPH$^{\bullet}$	HOCl	$O_2^{\bullet-}$
**PESE**	238.9 ± 1.9	14.5 ± 0.2^{c}	5.4 ± 0.5^{b}	13.9 ± 1.2^{b}
**PIC**	-	3.9 ± 0.1^{b} (15.9 µM)	0.5 ± 0.1^{a} (2.0 µM)	8.3 ± 0.3^{a} (34.2 µM)
**QUER**	-	1.4 ± 0.1^{a} (4.7 µM)	1.3 ± 0.2^{a} (4.4 µM)	21.7 ± 1.2^{c} (71.9 µM)

Mean ± SEM, n ≥ 3. Antioxidant assay data were analyzed using one-way ANOVA followed by Tukey's post hoc test. Within each assay, values with different superscript letters are significantly different ($p \leq 0.05$). TPC values were determined at a fixed concentration of 50 µg/mL, while antioxidant activities were expressed as IC_{50} (half-maximum inhibitory concentration, µg/mL). For reference, approximate IC_{50} values (µM) for pure compounds are provided in parentheses, calculated based on their respective molecular weights (piceatannol MW = 244.24 g/mol; quercetin MW = 302.24 g/mol); IC_{50} values for extracts are not expressed in µM due to their complex composition. TPC, Total phenol content; DE, dry extract; GAE, gallic acid equivalents; DPPH$^{\bullet}$, 2,2-diphenyl-1-picrylhydrazyl radical; $O_2^{\bullet-}$, superoxide anion radical; PESE, *Passiflora edulis* seed ethanolic extract; PIC, piceatannol; QUER, quercetin.

REFERENCES

Junior, F. C. d. S., Agues-Barbosa, T., Luchiari, A. C., & Medeiros, S. R. B. d. (2021). Genotoxicity and behavioral alterations induced by retene in adult zebrafish. *Journal of Environmental Chemical Engineering*, *9*(6), 106518. https://doi.org/10.1016/j.jece.2021.106518

Li, P., Li, Z., & Zhong, L. (2019). Effects of low concentrations of triphenyltin on neurobehavior and the thyroid endocrine system in zebrafish. *Ecotoxicol Environ Saf*, *186*, 109776. https://doi.org/10.1016/j.ecoenv.2019.109776
